# Development of a prediction method for severe pancreatitis using a nomogram

**DOI:** 10.3389/fmed.2026.1737122

**Published:** 2026-05-07

**Authors:** Wei Wei, Ying Wang, Pu Xie, Wei Wang, Song-guo Li, Shi-jie Lv, Ya-yun Hou, Dong Cui, Ren-ming Pei, Yun Zhu

**Affiliations:** 1 Department of Radiology, Anhui No. 2 Provincial People's Hospital, Hefei, Anhui, China; 2 Department of Ultrasound, Anhui No. 2 Provincial People's Hospital, Hefei, Anhui, China; 3 Department of Pathology, Anhui No. 2 Provincial People's Hospital, Hefei, Anhui, China; 4 Department of Radiology, The First Affiliated Hospital of Bengbu Medical University, Bengbu, Anhui, China

**Keywords:** area under the curve, nomogram, pancreatitis, prediction model, risk stratification

## Abstract

**Introduction:**

The identification of severe acute pancreatitis (SAP) is paramount for effective patient management, but the gold standard for diagnosing SAP requires over 48 h of organ failure, which may delay timely treatment. Hence, this study aimed to develop a prediction model for SAP using clinical characteristics, laboratory examinations, and non-contrast computed tomography (CT) signs.

**Methods:**

This retrospective study included patients admitted for acute pancreatitis between November 2019 and December 2025. The patients were randomized 7:3 to the training and internal validation sets. Patients from another hospital were included as the external validation set. The patients were grouped according to severity by the Atlanta classification. Selected features were used for the development and evaluation of a nomogram model for SAP prediction. The ROC curve, decision curve, and calibration curve were used for model performance evaluation.

**Results:**

A total of 1,128 patients (703, 300, and 125 patients in training, internal validation, and external validation sets) were included for analysis. The multivariable analysis revealed that age, diabetes, D-dimer, creatinine, serum calcium, WBC, VFR, CRP, decreased SpO_2_, ascites, and anterior renal fascia thickening were independently associated with SAP. The nomogram developed from the multivariable analysis demonstrated good performance for SAP prediction (AUC = 0.88, 95% confidence interval: 0.84–0.92) and exhibited excellent calibration abilities. Subgroup analysis among patients with hyperlipidemic or non-hyperlipidemic acute pancreatitis yielded similar results.

**Conclusion:**

A nomogram-based SAP prediction model may improve the capacity for risk stratification of pancreatitis severity. Exploratory subgroup analysis suggested the potential applications in patients with different causes.

## Introduction

Acute pancreatitis (AP) is an inflammatory disease of the pancreas most commonly caused by gallstones or excessive consumption of alcohol; although much effort has been spent on exploring the etiology and pathogenesis of AP, its underlying mechanisms remain controversial (1). AP is a potentially fatal gastroenterological disease that requires immediate treatment, especially severe AP (SAP). Annually, more than $2.6 billion is spent on the treatment of AP in the USA, with approximately 300,000 emergency department visits every year (2, 3).

According to the revised Atlanta classification, based on the disease course, the severity of AP can be categorized into mild, moderately severe, and severe (4). AP severity correlates with the mortality rate: 70%−75% of patients present with mild AP and have a very low mortality rate, 20%−25% have moderately SAP with increased mortality, and 5%−10% have SAP, which carries the highest mortality rate of approximately 15% (5). In recent years, several international expert panels have updated evidence-based guidelines to standardize the diagnosis, risk stratification, and management of AP, and all guidelines emphasize early identification of SAP, timely supportive care, and rational use of pharmacological interventions to reduce complications and mortality. These guidelines collectively highlight the importance of early warning indicators, such as clinical scoring systems and inflammatory biomarkers, for guiding clinical decision-making (6–8). Therefore, the early prediction of SAP is of critical importance. Although all AP patients generally receive similar supportive management at admission, including intravenous fluids, pain control, and fasting, early identification of those likely to develop SAP is essential for timely escalation of care. For instance, patients at risk for SAP may benefit from earlier transfer to intensive care, closer monitoring for organ failure, and proactive complication management. The current standard requiring >48 h of persistent organ failure to confirm SAP may delay these critical clinical decisions (4).

Although significant efforts have been made to predict the severity of pancreatitis early, accurate medical history and thorough physical examination remain central to clinical decision-making. Dobszai et al. (9) showed that body mass index (BMI) is a significant indicator of SAP, with a BMI >30 kg/m^2^ being associated with increased risk and mortality. In addition, Szentesi et al. (10) examined hypertension in the context of acute pancreatitis; however, unlike obesity, older age, or alcohol abuse, hypertension alone has not been consistently validated as an independent risk factor for SAP and is not included in most major severity scoring systems. These findings underscore the importance of clinical characteristics and their relationship to SAP risk.

In addition to clinical characteristics, laboratory parameters and imaging findings are also associated with AP severity. Shinzeki et al. (11) identified several risk factors for pancreatitis-related mortality, including base excess (BE), serum creatinine (Cr), blood sugar, and serum calcium; although aspartate aminotransferase (AST, formerly known as serum glutamate oxaloacetic transaminase) is often elevated in acute pancreatitis, it has not been established as a validated predictor of severity. Staubli et al. (12) found that the combination of C-reactive protein (CRP) and Pentraxin 3 (PTX3) yielded a prediction area under the curve (AUC) of 0.70. Furthermore, hyperlipidemic AP (HLAP), a subtype of AP, is primarily caused by abnormally elevated serum triglyceride levels in the blood (13). According to Nawaz et al. (14), patients with HLAP face a higher risk of developing complications and experiencing poor outcomes, highlighting hyperlipidemia as a significant contributor to AP severity.

From an imaging perspective, while contrast-enhanced computed tomography (CT) is regarded as the gold standard for diagnosing pancreatic necrosis (15), the disease's pathophysiology implies that necrosis usually does not occur within the first 48 h, limiting the utility of early contrast-enhanced CT. Consequently, non-contrast CT and other imaging modalities are gaining popularity. Lin et al. (16) demonstrated that a radiomics model outperformed conventional scoring systems for early discrimination, including the magnetic resonance (MR) severity index (MRSI), the Acute Physiology and Chronic Health Evaluation (APACHE) II, and the bedside index for severity in acute pancreatitis (BISAP). However, while MR provides multi-contrast imaging for severity assessment, it has limitations, including a scan time of up to 1 h and motion artifacts (17). In contrast, non-contrast CT is much faster, completing examinations with only one breath-hold. Additionally, it avoids the use of contrast agents, making it more suitable for patients with renal insufficiency or those at risk for contrast allergies (18).

Most previous prediction models have incorporated only a limited set of clinical, laboratory, and radiological features. Therefore, this study aimed to develop a nomogram-based model for predicting AP severity. A nomogram is a graphical tool used to visually approximate the value of a mathematical function. Nomograms are often used in medicine—for example, to calculate drug dosages based on height and weight or predict the risk of disease or recurrence (19).

## Materials and methods

### Study design and patients

This retrospective study included patients diagnosed with AP between November 2019 and December 2025 at the Anhui No. 2 Provincial People's Hospital. In addition, the validation set was from the Second Affiliated Hospital of Anhui Medical University. The inclusion criteria were (1) first occurrence of AP, diagnosed according to the revised 2012 Atlanta classification definition (4), (2) age ≥18 years, (3) abdominal non-contrast CT examinations were performed within 24 h of admission, and (4) completion of other relevant laboratory tests within 24 h of admission. The exclusion criteria were patients with (1) imaging features of chronic pancreatitis or a history of pancreatitis, (2) pancreatic neoplasms or previous pancreatic surgery, (3) incomplete biochemical tests within 24 h or low-quality CT images, or (4) prior treatment for acute pancreatitis at other hospitals before admission.

Patients with post-ERCP pancreatitis and trauma-related pancreatitis were excluded due to their distinct pathophysiological mechanisms and clinical courses, which differ from those of more common etiologies such as gallstones or hyperlipidemia. Including such cases may have introduced heterogeneity that could bias model performance.

This study was approved by the Ethics Committees of Anhui No. 2 Provincial People's Hospital (approval ID: (R)2024-061) and The Second Affiliated Hospital of Anhui Medical University (approval ID: (R)SL-YX2024-127). Informed consent was waived by the Ethics Committee of Anhui No. 2 Provincial People's Hospital due to the retrospective nature of this study. Confidentiality was maintained during the study process. All data are kept on a password-protected, secure server. All data were anonymized once the data collection process was completed. This observational study was reported in accordance with the STROBE checklist.

AP was diagnosed according to the 2012 Revised Atlanta classification, requiring at least two of the following: typical abdominal pain, serum amylase or lipase ≥3 times the upper limit of normal, or characteristic imaging findings. After diagnosis, patients were categorized as having mild, moderately severe, or severe AP based on the presence and duration of organ failure and local or systemic complications, in line with the Revised Atlanta definitions. Mild AP was defined as no organ failure and no local or systemic complications; moderately severe AP as transient organ failure (<48 h) and/or local complications and/or exacerbation of comorbid disease; and severe AP as persistent organ failure (≥48 h) (4).

The patients were divided into the mild-to-moderate group and the severe group based on the severity of pancreatitis, according to the Atlanta classification (4). Mild AP is the most common form and is characterized by the absence of organ failure and local or systemic complications, which typically resolve within the first week. Moderately severe AP is defined by the presence of transient organ failure, local complications, or exacerbation of comorbid diseases. SAP, on the other hand, is characterized by persistent organ failure lasting more than 48 h.

A total of 1,003 patients with AP from Anhui No. 2 Provincial People's Hospital were randomly divided into a training cohort (703 cases) and a validation cohort (300 cases) at a ratio of 7:3. Meanwhile, 125 patients from the Emergency Department of the Second Affiliated Hospital of Anhui Medical University were enrolled as an external validation set.

### Data collection

All clinical data and laboratory examination results were obtained from the medical history system and medical records. The management of the patients at the study centers followed standard clinical guidelines for AP (3, 6, 7, 20). These assessments were part of the standardized initial evaluation for AP patients at the study centers.

The clinical data included sex, age, BMI, onset-to-admission time, mean arterial pressure (MAP), body temperature, heart rate, smoking history, alcohol consumption history, diabetes status, hypertension, history of intestinal obstruction, and prior gallbladder surgery. For further analysis, the patients were categorized into three age groups based on a previous study (21): ≤ 40, 41–60, and ≥60 years.

The laboratory examination results at admission (i.e., the first available measurements after admission) included procalcitonin, lipase, amylase, D-dimer, triglycerides, creatinine, blood urea nitrogen (BUN), serum calcium, blood glucose, white blood cell (WBC) count, neutrophils, lymphocytes, the neutrophil-to-lymphocyte ratio (NLR), neutrophil ratio, lactate dehydrogenase (LDH), and alanine aminotransferase (ALT). CRP was the first CRP measurement within 24 h of admission.

The retroperitoneal inflammation score (RPIS), visceral-to-subcutaneous fat ratio (VFR), and presence of abdominal distension, pneumonia, pleural effusion, ascites, fatty liver, cholecystitis, gallstones, and thickening of the anterior renal fascia were assessed through CT examinations. All non-contrast CT (NCCT) examinations were performed within 24 h of admission. The use of NCCT reflects the real-world emergency workflow at the participating tertiary centers and was not mandated by the study protocol. In these centers, an initial NCCT is commonly obtained in patients with acute, undifferentiated abdominal pain to support or confirm the diagnosis of AP when needed and, crucially, to exclude other potentially life-threatening conditions that can mimic AP (e.g., perforated viscus, bowel obstruction, mesenteric ischemia, or aortic dissection). Importantly, NCCT findings were not used to define AP severity; staging into mild, moderately severe, and severe AP strictly followed the Revised Atlanta classification. Each included patient underwent one abdominal NCCT at baseline; data on subsequent CT or MRI examinations during follow-up were not systematically recorded and were therefore not analyzed in this study. The baseline NCCT protocol consisted of a single-phase, standard-dose abdominal acquisition, without intravenous contrast administration. As a result, the imaging variables used in the prediction model were derived exclusively from this initial NCCT examination.

A high-resolution CT scanner (Optima CT520, GE Healthcare, Wisconsin, USA) was utilized for examination during the study period, conducted from the diaphragm dome to the ischium using the following parameters: slice thickness of 5 mm, helical pitch of 5 mm, tube current of 300 mA, tube voltage of 120 kV, matrix of 512 × 512, and volume CT dose index (CTDIvol) of 17.39 mGy. The NCCT protocol (CTDIvol approximately 17 mGy) represents a standard-dose abdominal CT protocol at the study centers and avoids iodinated contrast, thereby minimizing the risk of contrast-induced acute kidney injury in this acutely ill population.

**Figure 1:**
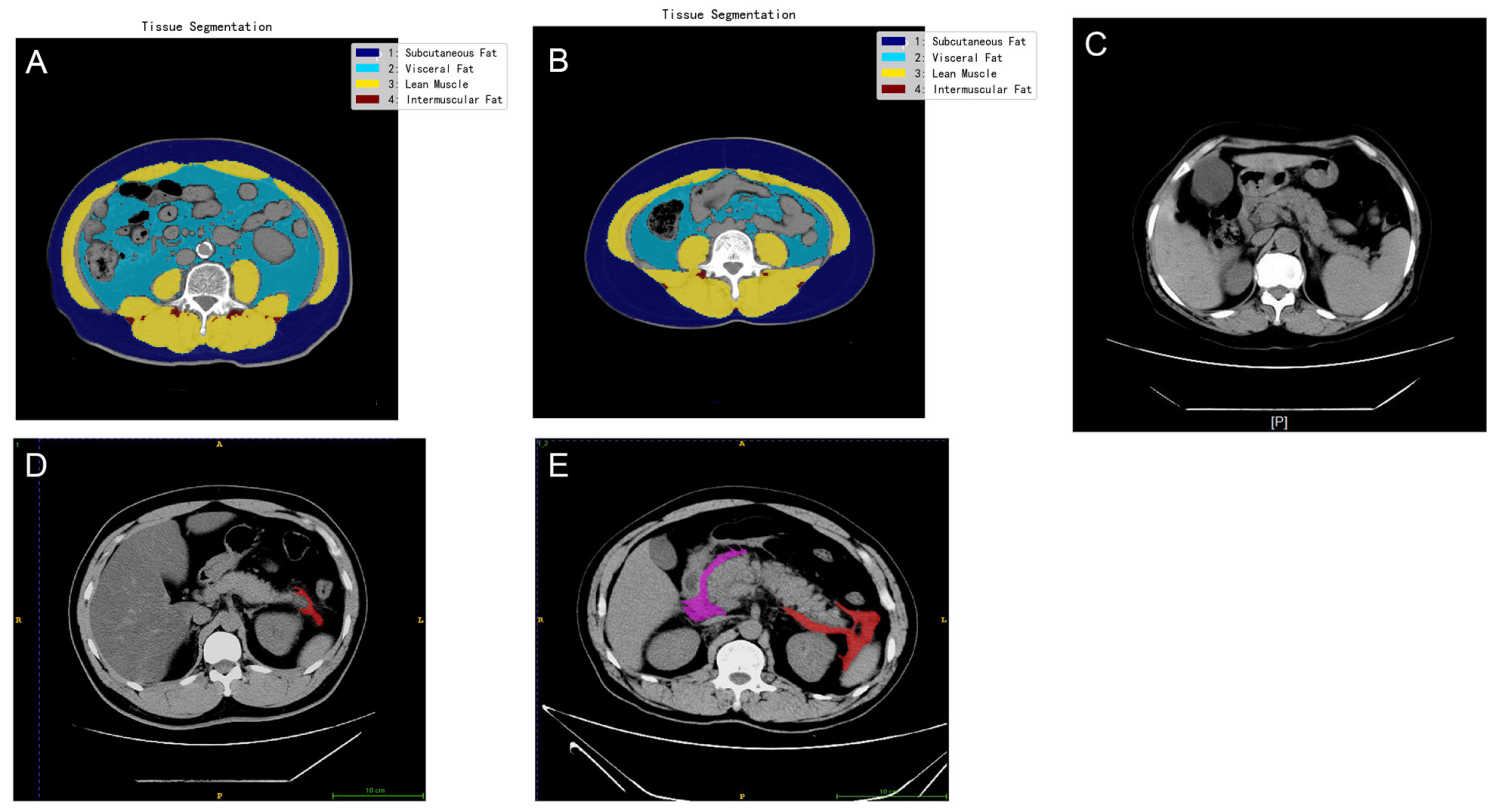
The non-enhanced computed tomography (CT) imaging for quantification of visceral fat and retroperitoneal inflammation. The inflammatory area is displayed in dark colors. **(A)** A 32-year-old male patient exhibited a visceral fat area of 116.389 cm^2^ and a subcutaneous fat area of 274.688 cm^2^, resulting in a visceral to subcutaneous fat ratio of 0.424. **(B)** A 58-year-old female patient had a visceral fat area of 64.035 cm^2^ and a subcutaneous fat area of 163.047 cm^2^, yielding a visceral to subcutaneous fat ratio of 0.393. **(C)** A patient without retroperitoneal inflammation. **(D)** A patient with left-sided retroperitoneal inflammation. **(E)** A patient with bilateral retroperitoneal inflammation.

The VFR was quantified at the midline level of the fourth lumbar vertebra using a deep learning-based automated body composition analysis system for CT images, which was dedicated to the quantitative assessment of muscular and adipose tissues at the spinal level with dual analyses of area and volume. For the deep learning-based segmentation of thoracoabdominal CT images, this system integrated TotalSegmentator (22) and nnU-Net (23), thereby enabling the automatic identification of three distinct adipose tissue components: subcutaneous fat, visceral fat, and intermuscular fat (Figures 1A, B). The RPIS was classified by a radiologist based on the CT images, with categories indicating none (0 points), unilateral (1 point), and bilateral inflammation (2 points) (Figures 1C–E). For other CT-derived variables (e.g., RPIS), 50 CT images were randomly selected and tested by two radiologists. No differences between the two radiologists were reported (Kappa = 1.00).

### Subgroup analysis

Exploratory subgroup analyses were performed. HLAP was diagnosed when the criteria for AP were met, accompanied by serum triglyceride (TG) levels of ≥1,000 mg/dl (≥11.30 mmol/L), or serum TG levels of 500–1,000 mg/dl (5.65–11.30 mmol/L) with the serum exhibiting a chylous appearance; additionally, other etiological factors of AP must be re-excluded (24). HLAP is considered the third most common cause of AP, following gallstones and chronic alcohol abuse. Compared with other types of AP, HLAP is associated with a more severe inflammatory response, a higher incidence of pancreatic cysts, and longer hospital stays. Consequently, a subgroup analysis based on this modified HLAP criteria was conducted to assess the performance of the included features, with all comparisons and regression analyses performed as previously described (16).

### Statistical analysis

All statistical analyses were conducted using R 4.3.1 (The R Project for Statistical Computing, https://www.r-project.org). The continuous data were tested for normal distribution using the Kolmogorov–Smirnov test. For continuous variables, those that were normally distributed were presented as mean ± standard deviation (SD) and compared using Student's *t*-test. Non-normally distributed continuous variables were reported as median (lower quartile, upper quartile) and analyzed with the Wilcoxon–Mann–Whitney test. Nominal data were expressed as *n* (%) and compared using the Chi-square test.

Univariable and multivariable logistic regression analyses were carried out to explore the associations between clinical characteristics and laboratory findings in SAP. Features with a *P*-value <0.20 in the univariable analyses were included in the stepwise multivariable regression analysis. The characteristics that showed quasi-complete separation (i.e., extreme value in univariable analyses) would not be included in the multivariable regression analysis. A nomogram was developed based on the results of the multivariable analysis. The prediction performance was assessed using the AUC, and the confidence interval (CI) of performance metrics was calculated by bootstrap with 500 iterations. Multicollinearity was tested for using the variance inflation factors (VIF), with VIF >5 indicating a risk of multicollinearity. For the subgroup analysis of HLAP, Firth's penalized logistic regression analysis was used due to the limited sample size.

A decision curve analysis (DCA) was conducted to evaluate the model's clinical usability, and a calibration curve was used to assess the model's generalizability. In this study, internal validation was performed using both the internal validation set and a bootstrap method with 500 iterations, and a two-tailed *P*-value <0.05 was considered statistically significant.

## Results

### Clinical characteristics and laboratory examinations

The study flowchart is shown in Supplementary Figure S1. Among the 1,003 patients from the training and internal validation sets, the mild-to-moderate group contained 875 patients (60.46% male), while the severe group contained 128 patients (58.59% male).

From the perspectives of clinical characteristics and CT signs, comparisons between the two groups revealed that patients with SAP had a higher percentages of intestinal obstruction (*P* = 0.012), exhibited significantly greater values for several factors: body temperature (*P* = 0.013), procalcitonin (*P* < 0.001), D-dimer (*P* < 0.001), CRP (*P* < 0.001), BUN (*P* = 0.005), WBC (*P* < 0.001), neutrophils (*P* < 0.001), NLR (*P* < 0.001), neutrophil ratio (*P* < 0.001), LDH (*P* < 0.001), blood glucose (*P* < 0.001), proportion of bilateral RPIS (*P* < 0.001), VFR (*P* < 0.001), decreased SpO_2_ (*P* < 0.001), pneumonia (*P* < 0.001), pleural effusion (*P* < 0.001), ascites (*P* < 0.001), cholecystitis (*P* < 0.001), and anterior renal fascia thickening (*P* < 0.001), and exhibited lower levels of serum calcium (*P* < 0.001) and lymphocytes (*P* < 0.001) (Table 1). Supplementary Table S1 shows that although there were differences in patient characteristics among the training, internal validation, and external validation sets, there were no significant differences in demographic characteristics and clinical history between the training and internal validation sets.

**Table 1:** Clinical characteristics, non-contrast CT signs, and laboratory examinations of all pancreatitis patients.

Variables	Total (*n* = 1,003)	Mild to moderate AP (*n* = 875)	Severe AP (*n* = 128)	Statistic	*P*-value
Demographic characteristics					
Sex, *n* (%)					
Female	399 (39.78)	346 (39.54)	53 (41.41)	χ^2^ = 0.09	0.803
Male	604 (60.22)	529 (60.46)	75 (58.59)	χ^2^ = 0.09	0.803
Age range, *n* (%)					
≤ 40	396 (39.48)	356 (40.69)	40 (31.25)	χ^2^ = 3.58	0.167
41–60	376 (37.49)	330 (37.71)	46 (35.94)		
>60	231 (23.03)	189 (21.60)	42 (32.81)		
Vital signs and BMI					
Body temperature, *M* (*Q*1, *Q*3)	36.50 (36.40, 36.60)	36.50 (36.40, 36.60)	36.50 (36.50, 36.60)	*Z* = 2.47	0.013
MAP, *M* (*Q*1, *Q*3)	98.33 (90.00, 107.67)	99.00 (90.50, 107.67)	96.00 (84.67, 106.83)	*Z* = 1.95	0.052
Heart rate, *M* (*Q*1, *Q*3)	85.00 (75.00, 100.00)	85.00 (75.00, 100.00)	88.50 (76.00, 102.25)	*Z* = 1.47	0.142
BMI, *M* (*Q*1, *Q*3)	24.75 (22.60, 27.11)	24.80 (22.60, 27.18)	24.34 (22.53, 26.69)	*Z* = 0.89	0.372
15-7.4,-1.3498ptClinical history					
Smoking history, *n* (%)					
No	632 (63.01)	553 (63.20)	79 (61.72)	χ^2^ = 0.05	0.821
Yes	371 (36.99)	322 (36.80)	49 (38.28)		
Alcohol history, *n* (%)					
No	610 (60.82)	542 (61.94)	68 (53.12)	χ^2^ = 3.28	0.070
Yes	393 (39.18)	333 (38.06)	60 (46.88)		
Diabetes, *n* (%)					
No	604 (60.22)	534 (61.03)	70 (54.69)	χ^2^ = 1.62	0.203
Yes	399 (39.78)	341 (38.97)	58 (45.31)		
Hypertension, *n* (%)					
No	581 (57.93)	511 (58.40)	70 (54.69)	χ^2^ = 0.49	0.485
Yes	422 (42.07)	364 (41.60)	58 (45.31)		
Hypertriglyceridemia medication history, *n* (%)					
No	675 (67.30)	586 (66.97)	89 (69.53)	χ^2^ = 0.23	0.634
Yes	328 (32.70)	289 (33.03)	39 (30.47)		
Abdominal distension, *n* (%)					
No	580 (57.83)	506 (57.83)	74 (57.81)	χ^2^ = 0.00	1.000
Yes	423 (42.17)	369 (42.17)	54 (42.19)		
Abdominal tenderness, *n* (%)					
No	240 (23.93)	203 (23.20)	37 (28.91)	χ^2^ = 1.70	0.193
Yes	763 (76.07)	672 (76.80)	91 (71.09)		
Intestinal obstruction, *n* (%)					
No	998 (99.50)	873 (99.77)	125 (97.66)	χ^2^ = 6.26	0.012
Yes	5 (0.50)	2 (0.23)	3 (2.34)		
Cholecystectomy history, *n* (%)					
No	925 (92.22)	809 (92.46)	116 (90.62)	χ^2^ = 0.35	0.554
Yes	77 (7.68)	65 (7.43)	12 (9.38)		
Onset to admission time, *M* (*Q*1, *Q*3)	13.00 (7.00, 28.50)	13.00 (7.00, 48.00)	12.00 (7.00, 24.00)	*Z* = 0.61	0.539
Procalcitonin, *M* (*Q*1, *Q*3)	0.15 (0.06, 0.41)	0.14 (0.06, 0.32)	0.33 (0.11, 1.67)	*Z* = 6.16	**< 0.001**
Lipase, *M* (*Q*1, *Q*3)	217.00 (78.00, 616.17)	213.05 (76.98, 587.75)	258.00 (96.30, 909.40)	*Z* = 1.81	0.071
Amylase, *M* (*Q*1, *Q*3)	147.50 (70.00, 565.50)	142.50 (72.25, 525.75)	182.50 (64.75, 688.00)	*Z* = 0.90	0.370
D-dimer, *M* (*Q*1, *Q*3)	1.17 (0.50, 2.17)	1.04 (0.46, 2.00)	2.74 (1.23, 6.84)	*Z* = 8.12	**< 0.001**
CRP, *M* (*Q*1, *Q*3)	41.29 (12.44, 115.13)	35.22 (10.94, 89.91)	134.72 (48.92, 201.62)	*Z* = 7.63	**< 0.001**
Triglyceride, *M* (*Q*1, *Q*3)	2.10 (1.04, 6.75)	2.06 (1.03, 6.68)	2.38 (1.06, 8.36)	*Z* = 0.79	0.427
Creatinine, *M* (*Q*1, *Q*3)	61.00 (50.00, 74.00)	60.00 (49.50, 73.00)	63.50 (50.00, 79.25)	*Z* = 1.85	0.064
BUN, *M* (*Q*1, *Q*3)	4.00 (3.10, 5.50)	4.00 (3.00, 5.40)	4.70 (3.27, 6.67)	*Z* = 2.81	**0.005**
Serum calcium, *M* (*Q*1, *Q*3)	2.16 (2.05, 2.26)	2.17 (2.07, 2.27)	2.09 (1.93, 2.23)	*Z* = 4.79	**< 0.001**
WBC, *M* (*Q*1, *Q*3)	9.62 (6.90, 12.94)	9.30 (6.66, 12.62)	11.40 (8.99, 14.47)	*Z* = 5.00	**< 0.001**
Neutrophils, *M* (*Q*1, *Q*3)	7.79 (4.92, 10.89)	7.28 (4.64, 10.58)	9.70 (7.84, 12.80)	*Z* = 6.07	**< 0.001**
Lymphocytes, *M* (*Q*1, *Q*3)	1.22 (0.85, 1.67)	1.25 (0.89, 1.70)	1.04 (0.68, 1.38)	*Z* = 4.36	**< 0.001**
NLR, *M* (*Q*1, *Q*3)	6.18 (3.35, 10.43)	5.64 (3.06, 9.61)	9.96 (5.74, 16.28)	*Z* = 7.19	**< 0.001**
Neutrophil ratio, *M* (*Q*1, *Q*3)	79.60 (69.10, 86.30)	78.30 (68.00, 85.20)	86.50 (78.52, 89.23)	*Z* = 7.24	**< 0.001**
LDH, *M* (*Q*1, *Q*3)	199.00 (158.41, 264.39)	195.93 (153.00, 255.87)	239.00 (184.00, 349.62)	*Z* = 5.18	**< 0.001**
ALT, *M* (*Q*1, *Q*3)	36.00 (20.00, 84.00)	36.00 (20.00, 87.00)	33.00 (19.00, 68.50)	*Z* = 1.12	0.264
Blood glucose, *M* (*Q*1, *Q*3)	7.35 (5.80, 10.57)	7.18 (5.71, 10.51)	8.34 (6.29, 11.74)	*Z* = 3.40	**< 0.001**
15-7.4,-1.3498ptCT signs					
RPIS, *n* (%)					
None	93 (9.27)	93 (10.63)	0 (0.00)	χ^2^ = 84.12	**< 0.001**
Unilateral	662 (66.00)	606 (69.26)	56 (43.75)		
Bilateral	247 (24.63)	175 (20.00)	72 (56.25)		
VFR, M (*Q*1, *Q*3)	0.85 (0.66, 1.18)	0.83 (0.64, 1.10)	1.06 (0.82, 1.46)	*Z* = 5.48	**< 0.001**
Decreased SpO_2_, *n* (%)					
No	987 (98.40)	872 (99.66)	115 (89.84)	–	**< 0.001**
Yes	16 (1.60)	3 (0.34)	13 (10.16)		
Pneumonia, *n* (%)					
No	524 (52.24)	493 (56.34)	31 (24.22)	χ^2^ = 45.09	**< 0.001**
Yes	478 (47.66)	381 (43.54)	97 (75.78)		
Pleural effusion, *n* (%)					
No	829 (82.65)	739 (84.46)	90 (70.31)	χ^2^ = 14.87	**< 0.001**
Yes	173 (17.25)	135 (15.43)	38 (29.69)		
Ascites, *n* (%)					
No	841 (83.85)	769 (87.89)	72 (56.25)	χ^2^ = 81.90	**< 0.001**
Yes	160 (15.95)	104 (11.89)	56 (43.75)		
Fatty liver, *n* (%)					
No	417 (41.58)	371 (42.40)	46 (35.94)	χ^2^ = 1.69	0.194
Yes	585 (58.33)	503 (57.49)	82 (64.06)		
Cholecystitis, *n* (%)					
No	697 (69.49)	624 (71.31)	73 (57.03)	χ^2^ = 10.34	**0.001**
Yes	304 (30.31)	249 (28.46)	55 (42.97)		
Gallstones, n (%)					
No	806 (80.36)	711 (81.26)	95 (74.22)	χ^2^ = 3.17	0.075
Yes	196 (19.54)	163 (18.63)	33 (25.78)		
Anterior renal fascia thickening, *n* (%)					
No	316 (31.51)	302 (34.51)	14 (10.94)	χ^2^ = 27.76	**< 0.001**
Yes	686 (68.39)	572 (65.37)	114 (89.06)		

ALT, alanine aminotransferase; BMI, body mass index; BUN, blood urea nitrogen; CRP, C-reactive protein; LDH, lactate dehydrogenase; MAP, mean arterial pressure; NLR, Neutrophil-to-Lymphocyte?Ratio; WBC, white blood cell.

The continuous data were not normally distributed and were presented as medians. Z: Mann-Whitney test, χ^2^: Chi-square test, −: Fisher exact; M: Median, Q_1_: 1st Quartile, Q_3_: 3rd Quartile.

Significant *P*-values are shown in bold.

### Multivariable analysis

All clinical characteristics and laboratory examinations underwent univariable analysis, many of which demonstrated an association with the severity of pancreatitis and were subsequently included in the multivariable analysis. The multivariable analysis revealed that age (OR: 1.026, 95% CI: 1.009–1.042, *P* = 0.002), diabetes (OR: 1.832, 95% CI: 1.040–3.225, *P* = 0.036), D-dimer (OR: 1.189, 95% CI: 1.102–1.283, *P* < 0.001), creatinine (OR: 1.009, 95% CI: 1.001–1.018, *P* = 0.024), serum calcium (OR: 0.178, 95% CI: 0.045–0.703, *P* = 0.014), WBC (OR: 1.095, 95% CI: 1.034–1.159, *P* = 0.002), VFR (OR: 1.860, 95% CI: 1.139–3.038, *P* = 0.013), CRP (OR: 1.004, 95% CI: 1.001–1.008, *P* = 0.009), decreased SpO_2_ (OR: 28.809, 95% CI: 5.379–154.292, *P* < 0.001), ascites (OR: 3.145, 95% CI: 1.688–5.858, *P* < 0.001), and anterior renal fascia thickening (OR: 2.332, 95% CI: 1.069–5.086, *P* = 0.033) were independently associated with SAP (Table 2). All VIFs were <5, indicating no multicollinearity (Supplementary Table S2).

**Table 2:** Univariable and multivariable analysis.

Variables	Univariable					Multivariable				
	β	SE	*Z*	*P*	OR (95%CI)	β	SE	*Z*	*P*	OR (95%CI)
Demographic characteristics										
Age	0.0208	0.0065	3.200	**0.002**	1.021 (1.008–1.034)	0.0253	0.0082	3.076	**0.002**	1.026 (1.009–1.042)
Gender										
Male					1.000 (Reference)					
Female	0.2522	0.2278	1.107	0.268	1.287 (0.823–2.011)					
Vital signs and BMI										
BMI	−0.0314	0.0311	−1.010	0.314	0.969 (0.912–1.03)					
MAP	−0.0177	0.0082	−2.159	**0.031**	0.982 (0.967–0.998)					
Heart rate	0.0037	0.006	0.617	0.546	1.004 (0.992–1.016)					
Body temperature	0.404	0.3063	1.319	0.187	1.498 (0.822–2.73)	0.5731	0.364	1.574	0.115	1.774 (0.869–3.621)
Clinical history										
Onset to admission time	−0.0036	0.003	−1.200	0.238	0.996 (0.991–1.002)					
Smoking history										
No					1.000 (Reference)					
Yes	−0.0016	0.2342	−0.007	0.994	0.998 (0.631–1.58)					
Alcohol history										
No					1.000 (Reference)					
Yes	0.3288	0.2281	1.441	0.150	1.389 (0.888–2.172)					
Diabetes										
No					1.000 (Reference)					1.000 (Reference)
Yes	0.4891	0.2266	2.158	**0.031**	1.631 (1.046–2.543)	0.6052	0.2887	2.096	**0.036**	1.832 (1.040–3.225)
Hypertension										
No					1.000 (Reference)					
Yes	0.1161	0.2274	0.511	0.610	1.123 (0.719–1.754)					
Hypertriglyceridemia medication history										
No					1.000 (Reference)					
Yes	−0.0184	0.2414	−0.076	0.939	0.982 (0.612–1.576)					
Abdominal distension										
No					1.000 (Reference)					
Yes	−0.1529	0.2301	−0.664	0.506	0.858 (0.547–1.347)					
Abdominal tenderness										
No					1.000 (Reference)					
Yes	−0.0983	0.2636	−0.373	0.709	0.906 (0.541–1.519)					
Intestinal obstruction										
No					1.000 (Reference)					
Yes	3.0494	1.1604	2.628	**0.009**	21.103 (2.171–205.146)					
Cholecystectomy history										
No					1.000 (Reference)					
Yes	0.2082	0.4015	0.519	0.604	1.231 (0.561–2.705)					
Biochemical markers										
Procalcitonin	0.1125	0.0315	3.571	**<0.001**	1.119 (1.052–1.190)					
Lipase	3e−04	1e−04	3.000	0.054	1.000 (1.000–1.001)					
Amylase	2e−04	1e−04	2.000	0.107	1.000 (1.000–1.000)					
D-dimer	0.2449	0.0327	7.489	**<0.001**	1.278 (1.198–1.362)	0.1732	0.0386	4.486	**<0.001**	1.189 (1.102–1.283)
CRP	0.008	0.0013	6.154	**<0.001**	1.008 (1.006–1.010)	0.0045	0.0017	2.621	**0.009**	1.004 (1.001–1.008)
Triglyceride	0.0244	0.0126	1.937	0.052	1.025 (1.000–1.050)					
Creatinine	0.014	0.0033	4.242	**<0.001**	1.014 (1.008–1.021)	0.0093	0.0041	2.256	**0.024**	1.009 (1.001–1.018)
BUN	0.1906	0.0421	4.527	**<0.001**	1.21 (1.114–1.314)					
Serum calcium	−3.1389	0.5626	−5.579	**<0.001**	0.043 (0.014–0.131)	−1.7239	0.6997	−2.464	**0.014**	0.178 (0.045–0.703)
WBC	0.0957	0.0229	4.179	**<0.001**	1.1 (1.052–1.151)	0.0906	0.0292	3.097	**0.002**	1.095 (1.034–1.159)
Neutrophils	0.1162	0.0242	4.802	**<0.001**	1.123 (1.071–1.178)					
Lymphocytes	−0.5637	0.1937	−2.910	**0.004**	0.569 (0.389–0.832)					
NLR	0.0517	0.0122	4.238	**<0.001**	1.053 (1.028–1.079)					
Neutrophil ratio	0.0657	0.0131	5.015	**<0.001**	1.068 (1.041–1.096)					
LDH	9e−04	4e−04	2.250	**0.049**	1.001 (1–1.002)	−0.0013	9e−04	−1.492	0.136	0.999 (0.997–1.000)
ALT	−2e−04	8e−04	−0.250	0.807	1.000 (0.998–1.001)					
Blood glucose	0.0504	0.0213	2.366	**0.018**	1.052 (1.009–1.097)					
CT signs										
VFR	0.7784	0.2037	3.821	**<0.001**	2.178 (1.461–3.246)	0.6206	0.2503	2.479	**0.013**	1.860 (1.139–3.038)
Decreased SpO_2_										
No					1.000 (Reference)					1.000 (Reference)
Yes	3.6425	0.7837	4.648	**<0.001**	38.187 (8.22–177.411)	3.3607	0.8562	3.925	**<0.001**	28.809 (5.379–154.292)
Pneumonia										
No					1.000 (Reference)					
Yes	1.2213	0.249	4.905	**<0.001**	3.392 (2.082–5.526)					
Pleural effusion										
No					1.000 (Reference)					
Yes	0.6916	0.2597	2.663	**0.008**	1.997 (1.2–3.322)					
Ascites										
No					1.000 (Reference)					1.000 (Reference)
Yes	1.8255	0.2472	7.385	**<0.001**	6.206 (3.822–10.075)	1.1457	0.3174	3.61	**<0.001**	3.145 (1.688–5.858)
Fatty liver										
No					1.000 (Reference)					
Yes	0.1395	0.2312	0.603	0.546	1.150 (0.731–1.809)					
Cholecystitis										
No					1.000 (Reference)					
Yes	0.8688	0.2295	3.786	**<0.001**	2.384 (1.520–3.738)					
Gallstones										
No					1.000 (Reference)					
Yes	0.4434	0.2597	1.707	0.088	1.558 (0.936–2.592)					
Anterior renal fascia thickening										
No					1.000 (Reference)					1.000 (Reference)
Yes	1.3342	0.3328	4.009	**<0.001**	3.797 (1.978–7.290)	0.8466	0.3979	2.127	**0.033**	2.332 (1.069–5.086)
RPIS										
None					1.000 (Reference)					
Unilateral	2.4706	NA		**0.007**	11.83 (1.65–1,502.16)	—	—	—	—	—
Bilateral	3.9142	NA		**<0.001**	50.111 (6.971–6366.48)	—	—	—	—	—

ALT, alanine aminotransferase; BMI, body mass index; BUN, blood urea nitrogen; CRP, C-reactive protein; LDH, lactate dehydrogenase; MAP, mean arterial pressure; NLR, Neutrophil-to-Lymphocyte?Ratio; WBC, white blood cell.

Significant *P*-values are shown in bold.

The full model equation was logit(*p*) = −24.725789 [Intercept] + 0.025290 × age + 0.573109 × temp + 0.173171 × D_dimer + 0.004463 × CRP + 0.009301 × creatinine – 1.723868 × calcium + 0.090572 × WBC – 0.001310 × LDH + 0.620594 × VFR + 3.360684 × SpO2_low + 0.605218 × diabetes + 1.145716 × ascites + 0.846566 × renal_fascia. For example, for a 55-year-old patient without decreased SpO_2_, diabetes, or anterior renal fascia thickening but with ascites and temperature of 37.8 °C, D-dimar at 2.5 mg/L, CRP at 150 mg/L, creatinine at 88 μmol/L, calcium at 2.1 mmol/L, WBC at 14 × 10^9^/L, LDH at 280 U/L, and VFR at 1.8, the model would yield a probability of SAP of 45.1%.

### Nomogram performance

**Figure 2:**
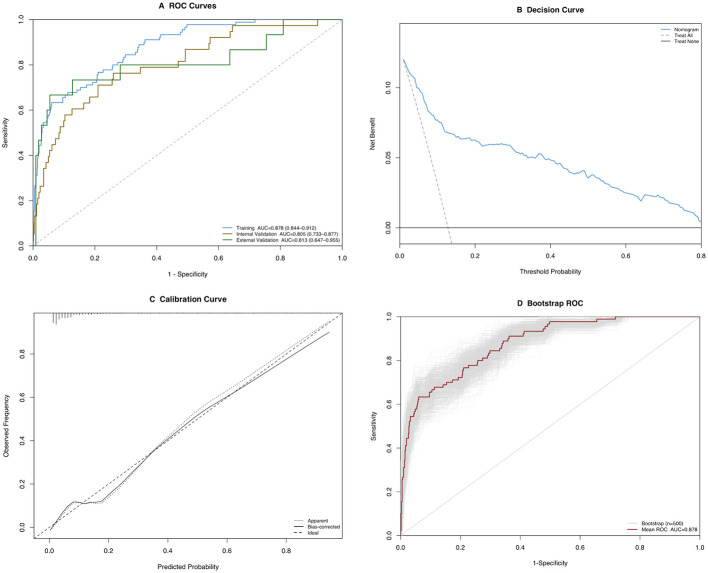
Model performance with all pancreatitis patients. **(A)** The receiver operating characteristics (ROC) curve for training and validation sets, **(B)** the decision curve analysis (DCA) curve, **(C)** the calibration curve, and **(D)** the ROC curve with confidence index in internal validation using bootstrap analysis.

We conducted a stepwise multivariable analysis, which identified several key features for nomogram development: age, temperature, D-dimer, CRP, creatinine, calcium, WBC, LDH, VFR, SpO_2_, diabetes, ascites, and renal fascia thickening (Supplementary Figure S2). The resulting nomogram, based on these features, demonstrated an AUC of 0.88 (95%CI: 0.84–0.92), with a sensitivity of 0.63 (95%CI: 0.53–0.72), specificity of 0.94 (95%CI: 0.92–0.96), accuracy of 0.90 (95%CI: 0.88–0.92), positive predictive value (PPV) of 0.61 (95%CI: 0.52–0.70), and negative predictive value (NPV) of 0.95 (95%CI: 0.93–0.96). In the internal validation set, the AUC was 0.81 (95%CI: 0.73–0.88) (Figure 2A). The external validation set showed an AUC of 0.81 (95%CI: 0.66–0.96, sensitivity of 0.67 (95%CI: 0.43–0.92), specificity of 0.95 (95%CI: 0.90–0.98), accuracy of 0.91 (95%CI: 0.86–0.96), PPV of 0.63 (95%CI: 0.38–0.85), and NPV of 0.95 (95%CI: 0.91–0.99). The DCA indicated a positive net benefit from the nomogram (Figure 2B). In addition, the calibration curve showed excellent performance (Figure 2C). Bootstrap internal validation also confirmed strong model performance, yielding an average AUC of 0.88 (Figure 2D). These results indicate that the nomogram based on the identified features performed well and demonstrated strong generalization and stability. In the training set, the nomogram's performance (AUC = 0.88, 95%CI: 0.85–0.91) was similar to that of APACHE II (AUC = 0.89, 95%CI: 0.87–0.92), BISAP (AUC = 0.88, 95%CI: 0.85–0.92), and RANSON (AUC = 0.87, 95%CI: 0.84–0.91). The nomogram generally showed lower performance in the internal and external validation sets (AUC = 0.851, 95%CI: 0.73–0.89, and AUC = 0.81, 95%CI: 0.64–0.97) compared with APACHE II (AUC = 0.90, 95%CI: 0.87–0.94, and AUC = 0.95, 95%CI: 0.89–0.99) but not worse than BISAP (AUC = 0.85, 95%CI: 0.77–0.91, and AUC = 0.66, 95%CI: 0.54–0.80) and RANSON (AUC = 0.86, 95%CI: 0.81–0.90, and AUC = 0.81, 95%CI: 0.70–0.91) (Table 3).

**Table 3:** Performance comparison: nomogram vs. BISAP, APACHE II, and RANSON across all datasets.

Score	Dataset	*N*	SAP_n	AUC	Sensitivity	Specificity	PPV	NPV	Accuracy	DeLong_p
APACHEII	External validation	125	15	0.95 (0.89–0.99)	0.80 (0.57–1.00)	0.97 (0.94–1.00)	0.80 (0.56–1.00)	0.97 (0.95–1.00)	0.95 (0.91–0.98)	0.058
BISAP	External validation	125	15	0.66 (0.54–0.80)	0.47 (0.25–0.73)	0.82 (0.74–0.89)	0.26 (0.13–0.42)	0.92 (0.87–0.97)	0.78 (0.70–0.86)	0.040
Our model	External validation	125	15	0.81 (0.64–0.97)	0.67 (0.43–0.94)	0.95 (0.89–0.98)	0.63 (0.36–0.83)	0.95 (0.92–0.99)	0.91 (0.86–0.96)	NA
RANSON	External validation	124	15	0.81 (0.70–0.91)	0.93 (0.77–1.00)	0.50 (0.40–0.59)	0.20 (0.125–0.30)	0.98 (0.94–1.00)	0.55 (0.46–0.63)	0.945
APACHEII	Internal validation	300	38	0.90 (0.87–0.94)	0.95 (0.87–1.00)	0.79 (0.73–0.84)	0.39 (0.29–0.47)	0.99 (0.98–1.00)	0.81 (0.76–0.85)	0.018
BISAP	Internal validation	300	38	0.85 (0.77–0.91)	0.63 (0.49–0.77)	0.95 (0.92–0.98)	0.63 (0.47–0.79)	0.95 (0.92–0.97)	0.91 (0.87–0.94)	0.415
Our model	Internal validation	300	38	0.81 (0.73–0.89)	0.76 (0.61–0.90)	0.74 (0.68–0.79)	0.30 (0.21–0.38)	0.96 (0.93–0.99)	0.74 (0.69–0.79)	NA
RANSON	Internal validation	300	38	0.86 (0.81–0.90)	0.97 (0.90–1.00)	0.60 (0.55–0.64)	0.26 (0.19–0.34)	0.99 (0.98–1.00)	0.64 (0.59–0.69)	0.256
APACHEII	Training	703	90	0.89 (0.87–0.92)	0.91 (0.85–0.97)	0.77 (0.74–0.80)	0.37 (0.31–0.43)	0.98 (0.97–0.99)	0.79 (0.76–0.82)	0.522
BISAP	Training	703	90	0.88 (0.85–0.92)	0.74 (0.66–0.83)	0.94 (0.93–0.96)	0.66 (0.57–0.73)	0.96 (0.95–0.98)	0.92 (0.90–0.93)	0.804
Our model	Training	703	90	0.88 (0.85–0.91)	0.63 (0.53–0.72)	0.94 (0.92–0.96)	0.61 (0.52–0.69)	0.95 (0.93–0.96)	0.90 (0.88–0.92)	NA
RANSON	Training	703	90	0.87 (0.84–0.91)	0.76 (0.67–0.84)	0.87 (0.85–0.90)	0.46 (0.40–0.55)	0.96 (0.95–0.98)	0.86 (0.83–0.88)	0.874

DeLong_p: *P*-value from the DeLong test comparing each score AUC against our model. APACHE II showed higher AUC in internal validation (0.904 vs. 0.805, *P* = 0.018); APACHE II requires ≥15 parameters assessed over 24 h in ICU and is not AP-specific. Our model significantly outperformed BISAP in external validation (0.813 vs. 0.660, *P* = 0.040). AUC, area under the curve; BISAP, Bedside Index for Severity in Acute Pancreatitis.

### Subgroup analysis

In order to explore the predictive value of clinical characteristics and laboratory examinations, all patients with AP were divided into two subgroups based on the underlying pathophysiological mechanisms for exploratory subgroup analyses: HLAP and other pancreatitis.

#### HLAP

Among 278 patients with HLAP, 43 developed SAP. Table 4 presents a comparison of clinical characteristics, non-contrast CT signs, and laboratory results. Statistically significant differences were observed in several laboratory markers (procalcitonin, D-dimer, CRP, serum calcium, WBC, neutrophils, NLR, neutrophil ratio, and LDH). Multivariable analysis identified several significant risk factors for severe HLAP, including ALT (OR: 1.009, 95% CI: 1.002–1.026, *P* = 0.010), serum calcium (OR: 0.021, 95% CI: 0.002–0.151, *P* < 0.001), and pneumonia (OR: 2.997, 95% CI: 1.212–8.877, *P* = 0.017) (Supplementary Table S3). The nomogram, formula was logit(*p*) = 5.002730 [Intercept] + 1.097537 × pneumonia + 0.017149 × BMI + 0.009048 × ALT + 0.031651 × PCT – 0.000276 × lipase – 3.882443 × calcium.

**Table 4:** Clinical characteristics, non-contrast CT signs, and laboratory examinations of hyperlipidemic pancreatitis patients.

Variables	Total (*n* = 277)	Mild to moderate HLAP (*n* = 234)	Severe HLAP (*n* = 43)	Statistic	*P*-value
Demographic characteristics					
Gender, *n* (%)					
Male	209 (75.5)	178 (76.1)	31 (72.1)	χ^2^ = 0.13	0.716
Female	68 (24.5)	56 (23.9)	12 (27.9)		
Age, *M* (*Q*1, *Q*3)	37.00 (32.00, 46.00)	36.50 (32.00, 45.75)	41.00 (32.00, 46.00)	*Z* = 0.72	0.470
Vital signs and BMI					
Body temperature, *M* (*Q*1, *Q*3)	36.50 (36.50, 36.50)	36.50 (36.50, 36.50)	36.50 (36.50, 36.60)	*Z* = 2.29	**0.022**
MAP, *M* (*Q*1, *Q*3)	101.33 (93.33, 110.00)	101.50 (94.67, 110.25)	97.00 (88.83, 108.67)	*Z* = 1.91	0.056
Heart rate, *M* (*Q*1, *Q*3)	95.00 (80.00, 109.00)	95.00 (80.00, 110.00)	96.00 (84.00, 107.00)	*Z* = 0.11	0.915
BMI, *M* (*Q*1, *Q*3)	25.69 (23.39, 28.26)	25.67 (23.42, 28.04)	26.04 (22.86, 29.53)	*Z* = 0.23	0.819
Clinical history					
Smoking history, *n* (%)					
No	157 (56.7)	133 (56.8)	24 (55.8)	χ^2^ = 0.00	1.000
Yes	120 (43.3)	101 (43.2)	19 (44.2)		
Alcohol history, *n* (%)					
No	157 (56.7)	133 (56.8)	24 (55.8)	χ^2^ = 0.00	1.000
Yes	120 (43.3)	101 (43.2)	19 (44.2)		
Diabetes, *n* (%)					
No	141 (50.9)	117 (50.0)	24 (55.8)	χ^2^ = 0.29	0.593
Yes	136 (49.1)	117 (50.0)	19 (44.2)		
Hypertension, *n* (%)					
No	149 (53.8)	125 (53.4)	24 (55.8)	χ^2^ = 0.01	0.902
Yes	128 (46.2)	109 (46.6)	19 (44.2)		
Hypertriglyceridemia medication history, *n* (%)					
No	148 (53.4)	121 (51.7)	27 (62.8)	χ^2^ = 1.38	0.241
Yes	129 (46.6)	113 (48.3)	16 (37.2)		
Abdominal distension, *n* (%)					
No	158 (57.0)	132 (56.4)	26 (60.5)	χ^2^ = 0.11	0.744
Yes	119 (43.0)	102 (43.6)	17 (39.5)		
Abdominal tenderness, *n* (%)					
No	49 (17.7)	41 (17.5)	8 (18.6)	χ^2^ = 0.00	1.000
Yes	228 (82.3)	193 (82.5)	35 (81.4)		
Intestinal obstruction, *n* (%)					
No	276 (99.6)	234 (100.0)	42 (97.7)	–	0.340
Yes	1 (0.4)	0 (0.0)	1 (2.3)		
Cholecystectomy history, *n* (%)					
No	263 (94.9)	223 (95.3)	40 (93.0)	–	0.805
Yes	14 (5.1)	11 (4.7)	3 (7.0)		
Onset to admission time, *M* (*Q*1, *Q*3)	12.00 (9.00, 24.00)	13.00 (9.00, 24.00)	12.00 (9.00, 24.00)	*Z* = 1.63	0.104
Biochemical markers					
Procalcitonin, *M* (*Q*1, *Q*3)	0.15 (0.07, 0.27)	0.14 (0.06, 0.22)	0.30 (0.15, 1.18)	*Z* = 4.46	<0.001
Lipase, *M* (*Q*1, *Q*3)	186.93 (77.90, 365.00)	180.50 (73.84, 300.00)	223.00 (116.66, 449.60)	*Z* = 1.32	0.186
Amylase, *M* (*Q*1, *Q*3)	111.00 (58.00, 283.00)	108.50 (59.25, 255.75)	168.00 (57.50, 548.00)	*Z* = 1.74	0.082
D-dimer, *M* (*Q*1, *Q*3)	1.14 (0.61, 1.93)	1.14 (0.50, 1.60)	2.03 (1.14, 4.97)	*Z* = 4.20	**<0.001**
CRP, *M* (*Q*1, *Q*3)	64.77 (26.42, 151.20)	52.70 (24.14, 137.00)	159.47 (63.05, 208.74)	*Z* = 3.84	**<0.001**
Triglyceride, *M* (*Q*1, *Q*3)	11.45 (8.12, 19.12)	11.28 (8.08, 18.84)	12.75 (8.59, 21.67)	*Z* = 0.75	0.453
Creatinine, *M* (*Q*1, *Q*3)	59.00 (48.00, 71.00)	59.00 (48.00, 71.00)	57.00 (47.00, 74.00)	*Z* = 0.14	0.891
BUN, *M* (*Q*1, *Q*3)	3.90 (2.90, 5.30)	3.80 (2.92, 5.38)	4.00 (3.05, 5.15)	*Z* = 0.14	0.890
Serum calcium, *M* (*Q*1, *Q*3)	2.17 (2.04, 2.28)	2.19 (2.07, 2.28)	2.06 (1.83, 2.19)	*Z* = 4.48	**<0.001**
WBC, *M* (*Q*1, *Q*3)	11.03 (8.01, 13.68)	10.25 (7.65, 13.54)	12.94 (10.78, 15.55)	*Z* = 3.72	**<0.001**
Neutrophils, *M* (*Q*1, *Q*3)	8.70 (5.77, 11.33)	8.27 (5.52, 10.85)	10.62 (8.96, 12.36)	*Z* = 4.19	**<0.001**
Lymphocytes, *M* (*Q*1, *Q*3)	1.41 (1.10, 1.83)	1.42 (1.11, 1.83)	1.21 (0.98, 1.82)	*Z* = 1.66	0.097
NLR, *M* (*Q*1, *Q*3)	5.62 (3.57, 8.97)	5.33 (3.15, 8.17)	9.32 (4.99, 11.65)	*Z* = 4.27	**<0.001**
Neutrophil ratio, *M* (*Q*1, *Q*3)	79.30 (71.50, 85.10)	78.35 (69.48, 84.40)	85.50 (77.85, 87.70)	*Z* = 4.21	**<0.001**
LDH, *M* (*Q*1, *Q*3)	192.00 (153.00, 244.00)	182.57 (147.25, 232.00)	261.80 (205.00, 432.12)	*Z* = 5.59	**<0.001**
ALT, *M* (*Q*1, *Q*3)	29.00 (19.00, 45.00)	28.00 (18.25, 42.75)	30.00 (19.50, 51.00)	*Z* = 0.91	0.365
Blood glucose, *M* (*Q*1, *Q*3)	9.89 (6.82, 14.08)	10.00 (6.80, 13.97)	8.85 (7.37, 14.43)	*Z* = 0.08	0.934
CT signs					
RPIS, *n* (%)				–	**<0.001**
VFR, M (Q1, Q3)	0.92 (0.72, 1.26)	0.90 (0.71, 1.20)	1.10 (0.91, 1.50)	*Z* = 3.16	**0.002**
Pneumonia, *n* (%)					
No	161 (58.1)	151 (64.5)	10 (23.3)	χ^2^ = 23.76	**<0.001**
Yes	116 (41.9)	83 (35.5)	33 (76.7)		
Pleural effusion, *n* (%)					
No	242 (87.4)	206 (88.0)	36 (83.7)	χ^2^ = 0.28	0.594
Yes	35 (12.6)	28 (12.0)	7 (16.3)		
Ascites, *n* (%)					
No	238 (85.9)	207 (88.5)	31 (72.1)	χ^2^ = 6.75	**0.009**
Yes	39 (14.1)	27 (11.5)	12 (27.9)		
Fatty liver, *n* (%)					
No	41 (14.8)	35 (15.0)	6 (14.0)	χ^2^ = 0.00	1.000
Yes	236 (85.2)	199 (85.0)	37 (86.0)		
Cholecystitis, *n* (%)					
No	237 (85.6)	206 (88.0)	31 (72.1)	χ^2^ = 6.24	**0.013**
Yes	40 (14.4)	28 (12.0)	12 (27.9)		
Gallstones, *n* (%)					
No	256 (92.4)	218 (93.2)	38 (88.4)	–	0.437
Yes	21 (7.6)	16 (6.8)	5 (11.6)		
Anterior renal fascia thickening, *n* (%)					
No	91 (32.9)	82 (35.0)	9 (20.9)	χ^2^ = 2.67	0.102
Yes	186 (67.1)	152 (65.0)	34 (79.1)		

ALT, alanine aminotransferase; BMI, body mass index; BUN, blood urea nitrogen; CRP, C-reactive protein; LDH, lactate dehydrogenase; MAP, mean arterial pressure; NLR, Neutrophil-to-Lymphocyte?Ratio; WBC, white blood cell.

The continuous data were not normally distributed and were presented as medians. *Z*, Mann–Whitney test; χ^2^, Chi-square test; –, Fisher exact; *M*, Median; *Q*1, 1st Quartile; *Q*3, 3rd Quartile.

Significant *P*-values are shown in bold.

The nomogram model (Supplementary Figure S3) demonstrated strong performance, achieving an AUC of 0.80 (95%CI: 0.68–0.89), with sensitivity, specificity, accuracy, PPV, and NPV of 0.72 (95%CI: 0.567–0.875), 0.78 (95%CI: 0.718–0.837), 0.77 (95%CI: 0.722–0.830), 0.36 (95%CI: 0.255–0.469), and 0.94 (95%CI: 0.896–0.977), respectively. The DCA curve indicated a net benefit, and the calibration curve was well-fitted. Internal validation yielded an AUC of 0.82 (95%CI: 0.72–0.90), and the bootstrap internal validation showed an average AUC of 0.80 (Supplementary Figure S4).

#### Other pancreatitis

A total of 727 patients were diagnosed with non-HLAP, of whom 86 had SAP. A comparison of baseline clinical characteristics, laboratory findings, and non-contrast CT signs is shown in Table 5. In this subgroup, the multivariable analysis indicated that ascites (OR: 7.686, 95% CI: 4.106–14.387, *P* < 0.001), CRP (OR: 1.007, 95% CI: 1.004–1.011, *P* < 0.001), and VFR (OR: 2.699, 95% CI: 1.525–4.0778, *P* < 0.001) (Supplementary Table S4). The nomogram formula was logit(*p*) = −4.315791 [Intercept] + 2.039434 × ascites + 0.007290 × CRP + 0.001000 × ALT + 0.993050 × VFR.

**Table 5:** Clinical characteristics, non-contrast CT signs, and laboratory examinations of non-HLAP.

Variables	Total (*n* = 726)	Mild to moderate non-HLAP (*n* = 641)	Severe non-HLAP (*n* = 85)	Statistic	*P*-value
Demographic characteristics					
Gender, *n* (%)					
Male	395 (54.4)	351 (54.8)	44 (51.8)	χ^2^ = 0.16	0.686
Female	331 (45.6)	290 (45.2)	41 (48.2)		
Age, *M* (*Q*1, *Q*3)	51.00 (37.00, 65.00)	50.00 (36.00, 63.00)	58.00 (41.00, 76.00)	*Z* = 3.87	**<0.001**
Vital signs and BMI					
Body temperature, *M* (*Q*1, *Q*3)	36.50 (36.40, 36.60)	36.50 (36.40, 36.60)	36.50 (36.50, 36.60)	*Z* = 1.51	0.131
MAP, *M* (*Q*1, *Q*3)	97.00 (89.00, 107.00)	97.33 (89.33, 107.00)	95.33 (84.67, 106.00)	*Z* = 1.34	0.180
Heart rate, *M* (*Q*1, *Q*3)	82.00 (74.00, 95.75)	82.00 (74.00, 95.00)	85.00 (74.00, 99.00)	*Z* = 1.10	0.272
BMI, *M* (*Q*1, *Q*3)	24.39 (22.41, 26.73)	24.44 (22.41, 26.83)	24.21 (22.31, 26.06)	*Z* = 1.07	0.285
Clinical history					
Smoking history, *n* (%)					
No	475 (65.4)	420 (65.5)	55 (64.7)	χ^2^ = 0.00	0.978
Yes	251 (34.6)	221 (34.5)	30 (35.3)		
Alcohol history, *n* (%)					
No	453 (62.4)	409 (63.8)	44 (51.8)	χ^2^ = 4.14	**0.042**
Yes	273 (37.6)	232 (36.2)	41 (48.2)		
Diabetes, *n* (%)					
No	463 (63.8)	417 (65.1)	46 (54.1)	χ^2^ = 3.43	0.064
Yes	263 (36.2)	224 (34.9)	39 (45.9)		
Hypertension, *n* (%)					
No	432 (59.5)	386 (60.2)	46 (54.1)	χ^2^ = 0.92	0.338
Yes	294 (40.5)	255 (39.8)	39 (45.9)		
Hypertriglyceridemia medication history, *n* (%)					
No	527 (72.6)	465 (72.5)	62 (72.9)	χ^2^ = 0.00	1.000
Yes	199 (27.4)	176 (27.5)	23 (27.1)		
Abdominal distension, *n* (%)					
No	422 (58.1)	374 (58.3)	48 (56.5)	χ^2^ = 0.04	0.832
Yes	304 (41.9)	267 (41.7)	37 (43.5)		
Abdominal tenderness, *n* (%)					
No	191 (26.3)	162 (25.3)	29 (34.1)	χ^2^ = 2.59	0.108
Yes	535 (73.7)	479 (74.7)	56 (65.9)		
Intestinal obstruction, *n* (%)					
No	722 (99.4)	639 (99.7)	83 (97.6)	–	0.108
Yes	4 (0.6)	2 (0.3)	2 (2.4)		
Cholecystectomy history, *n* (%)					
No	663 (91.3)	587 (91.6)	76 (89.4)	χ^2^ = 0.21	0.645
Yes	63 (8.7)	54 (8.4)	9 (10.6)		
Onset to admission time, *M* (*Q*1, *Q*3)	13.50 (6.00, 48.00)	12.00 (6.00, 48.00)	24.00 (6.00, 48.00)	*Z* = 0.24	0.813
Biochemical markers					
Procalcitonin, *M* (*Q*1, *Q*3)	0.15 (0.07, 0.37)	0.15 (0.07, 0.31)	0.30 (0.12, 1.70)	*Z* = 4.61	**<0.001**
Lipase, *M* (*Q*1, *Q*3)	223.00 (86.89, 696.64)	223.00 (85.00, 672.00)	245.67 (97.24, 944.23)	*Z* = 1.51	0.130
Amylase, *M* (*Q*1, *Q*3)	186.50 (79.00, 749.75)	183.00 (80.00, 716.00)	204.00 (67.00, 1013.00)	*Z* = 0.36	0.716
D-dimer, *M* (*Q*1, *Q*3)	1.14 (0.60, 2.09)	1.14 (0.55, 1.93)	2.31 (1.14, 6.74)	*Z* = 6.85	**<0.001**
CRP, *M* (*Q*1, *Q*3)	37.63 (10.21, 86.72)	31.77 (9.19, 77.17)	119.76 (41.29, 185.19)	*Z* = 6.44	**<0.001**
Triglyceride, *M* (*Q*1, *Q*3)	1.34 (0.87, 2.44)	1.36 (0.87, 2.48)	1.23 (0.90, 2.38)	*Z* = 0.91	0.361
Creatinine, *M* (*Q*1, *Q*3)	61.00 (50.00, 75.00)	61.00 (50.00, 74.00)	67.00 (53.00, 80.00)	*Z* = 2.27	**0.023**
BUN, *M* (*Q*1, *Q*3)	4.10 (3.10, 5.60)	4.00 (3.00, 5.50)	4.90 (3.60, 7.00)	*Z* = 3.42	**0.001**
Serum calcium, *M* (*Q*1, *Q*3)	2.16 (2.06, 2.26)	2.16 (2.07, 2.26)	2.10 (1.96, 2.24)	*Z* = 2.69	**0.007**
WBC, *M* (*Q*1, *Q*3)	9.17 (6.61, 12.59)	8.96 (6.42, 12.40)	10.35 (7.85, 14.38)	*Z* = 3.30	**0.001**
Neutrophils, *M* (*Q*1, *Q*3)	7.22 (4.64, 10.73)	6.91 (4.51, 10.38)	9.27 (6.32, 12.85)	*Z* = 4.31	**<0.001**
Lymphocytes, *M* (*Q*1, *Q*3)	1.14 (0.78, 1.60)	1.18 (0.82, 1.64)	0.80 (0.62, 1.31)	*Z* = 4.70	**<0.001**
NLR, *M* (*Q*1, *Q*3)	6.42 (3.28, 11.47)	5.91 (2.99, 10.49)	10.90 (6.72, 17.37)	*Z* = 6.01	**<0.001**
Neutrophil ratio, *M* (*Q*1, *Q*3)	79.60 (68.53, 86.80)	78.30 (67.60, 85.70)	87.20 (79.10, 89.50)	*Z* = 5.96	**<0.001**
LDH, *M* (*Q*1, *Q*3)	203.80 (160.25, 276.00)	201.00 (157.00, 272.92)	237.00 (180.00, 306.00)	*Z* = 2.60	**0.009**
ALT, *M* (*Q*1, *Q*3)	41.00 (21.00, 114.75)	42.00 (21.00, 118.00)	34.00 (19.00, 88.00)	*Z* = 1.47	0.141
Blood glucose, *M* (*Q*1, *Q*3)	6.82 (5.55, 9.25)	6.72 (5.48, 8.92)	7.96 (6.28, 10.61)	*Z* = 3.71	**<0.001**
CT signs					
RPIS, *n* (%)				χ^2^ = 69.01	**<0.001**
VFR, *M* (*Q*1, *Q*3)	0.83 (0.61, 1.10)	0.81 (0.60, 1.08)	1.00 (0.77, 1.41)	*Z* = 4.33	**<0.001**
Pneumonia, *n* (%)					
No	364 (50.1)	343 (53.5)	21 (24.7)	χ^2^ = 23.77	**<0.001**
Yes	362 (49.9)	298 (46.5)	64 (75.3)		
Pleural effusion, *n* (%)					
No	588 (81.0)	534 (83.3)	54 (63.5)	χ^2^ = 17.81	**<0.001**
Yes	138 (19.0)	107 (16.7)	31 (36.5)		
Ascites, *n* (%)					
No	605 (83.3)	564 (88.0)	41 (48.2)	χ^2^ = 82.55	**<0.001**
Yes	121 (16.7)	77 (12.0)	44 (51.8)		
Fatty liver, *n* (%)					
No	376 (51.8)	336 (52.4)	40 (47.1)	χ^2^ = 0.66	0.416
Yes	350 (48.2)	305 (47.6)	45 (52.9)		
Cholecystitis, *n* (%)					
No	462 (63.6)	420 (65.5)	42 (49.4)	χ^2^ = 7.74	**0.005**
Yes	264 (36.4)	221 (34.5)	43 (50.6)		
Gallstones, *n* (%)					
No	551 (75.9)	494 (77.1)	57 (67.1)	χ^2^ = 3.58	0.058
Yes	175 (24.1)	147 (22.9)	28 (32.9)		
Anterior renal fascia thickening, *n* (%)					
No	225 (31.0)	220 (34.3)	5 (5.9)	χ^2^ = 27.07	**<0.001**
Yes	501 (69.0)	421 (65.7)	80 (94.1)		

ALT, alanine aminotransferase; BMI, body mass index; BUN, blood urea nitrogen; CRP, C-reactive protein; LDH, lactate dehydrogenase; MAP, mean arterial pressure; NLR, neutrophil-to-lymphocyte ratio; WBC, white blood cell.

The continuous data were not normally distributed and were presented as medians. *Z*, Mann–Whitney test; χ^2^, Chi-square test; –, Fisher exact; *M*, Median; *Q*1, 1st Quartile; *Q*3, 3rd Quartile.

Significant *P*-values are shown in bold.

The nomogram model (Supplementary Figure S5) demonstrated good predictive performance, with an AUC of 0.84 (95%CI: 0.78–0.89). The model achieved a sensitivity of 0.75 (95%CI: 0.64–0.85), specificity of 0.82 (95%CI: 0.79–0.85), accuracy of 0.81(95%CI: 0.78–0.85), PPV of 0.36 (95%CI: 0.23–0.44), and NPV of 0.96 (95%CI: 0.94–0.98). The DCA curve indicated a net benefit, and the calibration curve was well-fitted. Internal validation showed an AUC of 0.75 (95%CI: 0.64–0.84), and the bootstrap internal validation showed an average AUC of 0.84 (Supplementary Figure S6).

## Discussion

To date, no reliable tool is available for predicting moderately severe and severe AP at admission with a favorable positive predictive value. Notably, three landmark guideline statements have consistently emphasized that early and accurate risk stratification represents a cornerstone in the contemporary management of AP (6–8). Early and accurate identification of patients at risk of developing SAP may facilitate timely decision-making regarding patient triage, transfer, and therapeutic management, as well as rational subject enrollment and grouping in clinical research. Typical diagnosis of SAP necessitates continuous monitoring for at least 48 h. This study focused on clinical characteristics, CT signs, and laboratory examinations to develop a predictive model for AP severity using admission variables, allowing SAP prediction from admission and without the need for a 48-h observation. The nomogram demonstrated promising performance, achieving an AUC of 0.88. In addition to the overall predictive model, the study developed subgroup-specific nomograms for patients with HLAP and those with AP of other etiologies. These subgroup models also appear promising, with the HLAP nomogram achieving an AUC of 0.80, and the non-HLAP nomogram showing an AUC of 0.84. These findings suggest that tailored prediction tools based on underlying pathophysiology may enhance accuracy in clinical practice.

SAP has long been a challenging issue for patients with AP, with a poorer prognosis. Many available nomograms for SAP are limited to predicting mortality in patients with SAP (25–27), and do not predict SAP itself. The model developed here demonstrated an AUC of 0.88, which aligns with previous studies: AUC = 0.889 for predicting SAP (28) and AUC = 0.853–0.865 for predicting moderately severe and SAP in pregnancy (29). A study showed that procalcitonin, TG, white blood cells at 48 h, hematocrit at 48 h, and calcium at 48 h could predict necrotizing pancreatitis (AUC = 0.822) (30), but it was based on 48-h values. Two studies associated various factors with organ failure in AP (31, 32). Radiological examinations, including MRI and CT, are crucial tools for pancreatitis risk stratification. Ma et al. (33) developed a gallstone pancreatitis severity prediction model based on CT images and a machine learning algorithm, achieving a performance score of 0.914. Another study focused on the pediatric population using CT features, reporting a sensitivity of 71.4% and specificity of 72.2% (34). Regarding traditional scores, the nomogram achieved an AUC that compared favorably with the APACHE II, BISAP, and Ranson scores in the training set, but APACHE II performed better in the validation sets. APACHE II is an extensively validated score; its coefficients and structure have been optimized across multiple populations, which often leads to more stable performance when transported to new settings, even if a new model looks superior in its training cohort (35). Furthermore, the APACHE II score usually requires the worst measurement during the first 24 h after admission, whereas the variables included in our model were available at admission or at the first measurement, making it suitable for early decisions before a full ICU assessment is possible.

D-dimer is a feature retained in the nomogram model, and its effectiveness has been validated in the study by Wan et al. (36), which found that D-dimer levels >2.5 mg/L were associated with a higher incidence of SAP. Other features recognized in previous studies include amylase (37), CRP (38), presence of fatty liver (39), and presence of cholecystitis (40), all of which indicate that the present model effectively selected valuable features. In addition, the model identified novel features (such as age, diabetes, VFR, decreased SpO_2_, ascites, and anterior renal fascia thickening) for predicting SAP, which require further validation.

Subgroup analyses were performed, but due to the smaller numbers of patients and events, those analyses must be considered exploratory and interpreted with caution. Nevertheless, our subgroup analyses revealed differing risk factor profiles: in the non-HLAP subgroup, ascites, CRP, and VFR were independent risk factors for SAP, while in the HLAP subgroup, ALT, serum calcium, and pneumonia were significant. These discrepancies likely reflect the distinct pathophysiological mechanisms underlying different AP etiologies. In HLAP, metabolic derangements, including obesity-related systemic inflammation and triglyceride-induced pancreatic injury, may underpin the prominence of BMI, lipid-related enzymes, and calcium dysregulation in this subgroup. Indeed, high BMI has been independently associated with SAP and mortality in AP (41, 42), while HLAP patients are known to exhibit more severe inflammatory responses and comorbidities compared to other etiologies (43). Furthermore, in the context of hypertriglyceridemia-induced AP, low serum calcium and elevated BMI and inflammatory markers have been identified as key risk factors (44). This highlights the heterogeneous nature of AP etiologies and supports the clinical utility of tailored predictive models for different patient subsets.

In the present study, features such as pleural effusion and ascites revealed by non-enhanced CT examinations were selected. Compared with contrast-enhanced CT, non-contrast CT has several advantages: it is better tolerated by patients with renal impairment (45), and enhanced CT offers limited additional value in patients without pancreatic necrosis (20, 46). Although non-contrast CT has limited ability to discriminate soft tissue (47), it remains a mainstream tool for pancreatitis screening, diagnosis, and severity classification, with its performance widely accepted by clinicians. More importantly, compared with contrast-enhanced CT, non-contrast CT shows great potential for the timely evaluation of SAP at primary hospitals. Radiologists can calculate the nomogram-based score immediately after the examination, aiding clinicians in deciding whether patients should be transferred to specialized hospitals.

The present study suggested that VFR is a significant risk factor for AP. Visceral fat can trigger systemic inflammation through the release of inflammatory adipokines. This inflammation can contribute to various health problems, including pancreatitis (48, 49). AP can be exacerbated by systemic exacerbation from other sources (through circulating cytokines), contributing to severity progression (50–52). This finding is supported by a study by Singh et al. (53), which examined fat tissue in relation to AP. Their research revealed that biliary AP is linked to a markedly higher percentage of intrapancreatic fat. In addition, a study by Mery et al. (54) focused on android fat distribution, finding that patients with higher waist circumference and android fat distribution faced an increased risk of developing SAP. While these studies indicated that fat distribution may contribute to SAP, there is limited research specifically addressing VFR's role in this context. Sternby et al. (55) found no correlation between AP severity and either visceral or subcutaneous adipose tissue; however, they did not assess the relationship between VFR and pancreatitis. Conversely, Norbitt et al. (56) noted that VFR could be a predictor for the development of diabetes following AP. This study demonstrated significant correlations between HLAP severity and VAI, CMI, and LAP indicators. These indicators, particularly VAI, which displayed the highest predictive power, were instrumental in forecasting and evaluating the severity of HLAP (57). Unlike other fat distribution metrics, VFR provides a broader perspective on fat distribution patterns. In this study, VFR demonstrated the highest OR among all assessed factors. Although additional animal studies or large-scale cohort data are needed to validate these findings, our results suggest that clinicians should pay closer attention to visceral fat.

The VFR was associated with the risk of severe illness in the overall cohort and the non-HLAP subgroup, but not significantly in the HLAP subgroup. Of course, the small sample size can influence the associations. Nevertheless, in HLAP, pathogenesis is thought to be driven primarily by systemic lipotoxicity from elevated triglyceride-rich lipoproteins and their breakdown to free fatty acids (FFAs) within pancreatic microcirculation, which can cause acinar injury, endothelial dysfunction, microcirculatory stasis, and inflammation leading to necrosis and organ failure (16, 58, 59). In non-HLAP (e.g., biliary, alcohol), visceral adiposity appears to be a key modulator of AP severity, with higher VAT/VAT ratios linked to more severe disease, ICU admission, and complications (60–62). In HLAP, however, severity correlates more closely with triglyceride level, lipoprotein burden, and FFA-mediated lipotoxicity than with regional fat distribution *per se*, which may blunt the additional impact of VFR in multivariable models (16, 58, 59). Etiology can act as an effect modifier, such that the relationship between VFR and severe outcomes is strong in non-HTG patients but attenuated or null in those with HTG-AP (63).

Machine learning (ML) methods have advantages in capturing non-linear relationships and variable interactions, techniques that have been increasingly adopted in medical data science (64). The decision to use a nomogram based on logistic regression was carefully considered and primarily driven by the focus on clinical interpretability and practical applicability. Indeed, clinical interpretability is essential for facilitating the adoption of predictive tools in real-world settings. ML models are “black-box” systems with limited transparency, and the variables are often not observable by humans. On the other hand, a logistic regression-based nomogram based on clinical data provides a clear, quantitative depiction of each predictor's contribution to the outcome (65, 66). This transparency enables clinicians to understand, communicate, and trust the model's predictions, an indispensable feature for clinical integration and patient communication. Second, the goal was to develop a simple and reproducible tool suitable for broad clinical use, including in primary care or resource-limited settings (19). Firth's penalized logistic regression was used to account for the limited number of patients and events (67).

In the study centers, NCCT within 24 h of admission was part of the standardized initial evaluation for patients with suspected AP, mainly to confirm the diagnosis in selected cases and to rule out alternative causes of acute abdominal pain, rather than to assess necrosis or determine severity. This is consistent with imaging guidelines that discourage routine early CT in clearly mild AP but allow early imaging when the diagnosis is uncertain or when other serious conditions must be excluded (68, 69). Prior studies have shown that early NCCT could be useful in prognostic models in AP, including cohorts in which CT was performed within 24 h of admission. These findings suggest that early NCCT should be viewed as a pragmatic adjunct for initial risk assessment rather than a substitute for guideline-recommended delayed contrast-enhanced imaging when the latter is appropriate (70, 71). Nevertheless, the incorporation of NCCT-derived variables into the model may limit its generalizability in settings where early CT is not routinely performed, and the model should be interpreted with caution in such environments. Moreover, by relying on NCCT rather than contrast-enhanced CT in the very early phase, the study centers aimed to balance diagnostic clarity with patient safety, given concerns about the limited incremental prognostic value of early contrast-enhanced CT and the potential risk of contrast-induced kidney injury in necrotizing AP (68, 72).

An additional concern in the imaging evaluation of AP is the cumulative radiation dose associated with repeated CT examinations, particularly in patients with severe or necrotizing disease. Ball et al. (73) reported that patients with necrotizing pancreatitis received a median of five CT scans, with an average effective dose of approximately 40–63 mSv per patient, equivalent to about 2,000 chest radiographs and associated with an increased estimated lifetime risk of fatal cancer. Other studies similarly found that CT utilization and cumulative radiation exposure increase with AP severity and with the use of multiphase protocols (74, 75). In this context, the nomogram, built on a single early NCCT and routine clinical and laboratory parameters, may support more rational use of cross-sectional imaging: for patients predicted to be at low risk of severe AP, follow-up contrast-enhanced CT can potentially be reduced or deferred in the absence of clinical deterioration, whereas in high-risk patients, CT can be targeted to those most likely to benefit, thereby helping to balance diagnostic yield against radiation exposure.

Despite the promising predictive performance of our model, several limitations of this study should be acknowledged. First, the study was conducted based on data from only two centers, and the total sample size remains relatively limited, especially in subgroup analyses, leading to inadequate statistical power to detect potential differences in predictive efficacy among different subgroups. Furthermore, the early single non-contrast CT (NCCT) acquired within 24 h of admission strictly adhered to the standardized emergency workflow of the participating centers; this protocol was primarily implemented to exclude life-threatening abdominal mimics of acute pancreatitis (AP) and clarify atypical clinical or biochemical manifestations, rather than to establish the definitive diagnosis or severity stratification of AP. All enrolled patients were categorized according to the Revised Atlanta classification relying on clinical trajectories and organ failure assessment independent of initial NCCT findings, yet this localized imaging strategy is inherently non-generalizable to institutions without routine early emergency NCCT pathways, restricting the extrapolation of our model in such clinical scenarios. Additionally, only baseline admission NCCT data were analyzed in the current cohort, while subsequent repeated cross-sectional imaging examinations were not systematically collected, precluding comprehensive evaluation of cumulative radiation exposure associated with multiple follow-up CT/MRI scans during hospitalization; subtle inter-institutional discrepancies in the practical application of the Revised Atlanta criteria may further compromise the external validation performance of the developed predictive tool. Second, due to limitations in clinical data availability from the charts, several potential prognostic parameters recommended by relevant clinical guidelines (such as interleukin-6 [IL-6], hematocrit [HCT], bilirubin, and cholesterol) were not included in the model. Third, the manual measurement of VFR is inherently associated with subjective inter-observer and intra-observer errors. To mitigate this issue, a deep learning platform was introduced for auxiliary measurement; however, the accuracy and reliability of this newly applied deep learning tool have not been fully verified and validated against a gold standard method. Fourth, the subgroup stratification in this study was relatively simple: AP patients were merely divided into HLAP and non-HLAP subtypes, without a more refined classification. Fifth, this study adopted a retrospective study design, which inherently imposes certain limitations. Sixth, the model was constructed and validated based on data from tertiary medical centers, and its applicability in primary medical institutions and other clinical settings remains untested, which limits the generalization of the model to broader clinical scenarios. Finally, the model achieved high NPVs but low PPVs. Clinically, it means that a negative nomogram test can be used to exclude SAP, but a positive result should be taken with caution to remain vigilant for SAP development, while avoiding overtreatment. Hence, the limitations mentioned above should be taken into account when interpreting the results of this study. In future research, we will expand the sample size, include multicenter data from diverse clinical settings, supplement the missing prognostic parameters, verify and optimize the deep learning measurement platform, conduct refined subgroup analysis based on different etiological subtypes of AP, and design prospective studies to further improve the comprehensiveness, accuracy, and clinical applicability of the prediction model, thereby providing more reliable clinical decision-making support for the early severity assessment and personalized management of AP patients.

## Conclusions

This study created a nomogram-based model for predicting SAP. The proposed model demonstrated discrimination ability and generalizability. Exploratory subgroup analysis in the HLAP and non-HLAP subgroups suggested the potential applications in patients with different causes. This nomogram could help determine the risk of SAP and start treatments early, i.e., before waiting for the 48-h organ failure period for SAP confirmation. Still, the model must be taken with caution pending external validation and refinement.

## Data Availability

The original contributions presented in the study are included in the article/Supplementary material, further inquiries can be directed to the corresponding authors.

## Supplementary material

Supplementary Figure S1 Study inclusion flowchart.

Supplementary Figure S2 Nomogram for severe pancreatitis among all pancreatitis patients. MAP, mean arterial pressure; CRP 24h, C-reactive protein within 24 hours after admission.

Supplementary Figure S3 Nomogram for severe pancreatitis among hyperlipidemic acute pancreatitis patients. MAP, mean arterial pressure; BMI, body mass index; ALT, alanine aminotransferase.

Supplementary Figure S4 Model performance among hyperlipidemic acute pancreatitis patients. **(A)** ROC curve; **(B)** DCA curve; **(C)** calibration curve; **(D)** ROC curve in bootstrap internal validation.

Supplementary Figure S5 Nomogram for severe pancreatitis among non-hyperlipidemic acute pancreatitis patients. BMI, body mass index; CRP 24h, C-reactive-protein within 24 h after admission; ALT, alanine aminotransferase.

Supplementary Figure S6 Model performance and evaluation with non-hyperlipidemic acute pancreatitis patients. **(A)** ROC curve; **(B)** DCA curve; **(C)** calibration curve; **(D)** ROC curve in bootstrap internal validation.

Supplementary Table S1 Comparison of the training, internal validation, and external validation sets.

Supplementary Table S2 Multicollinearity assessment.

Supplementary Table S3 Univariable and multivariable analysis for the development of severe pancreatitis in hyperlipidemic acute pancreatitis patients.

Supplementary Table S4 Univariable and multivariable analysis for the development of severe pancreatitis in non-hyperlipidemic acute pancreatitis patients.
