# Supplementary figures and images for: Development of a prediction method for severe pancreatitis using a nomogram
Source: Front Med (Lausanne). 2026 May 7;13:1737122. doi: 10.3389/fmed.2026.1737122 (PMC13190413; doi:10.3389/fmed.2026.1737122)

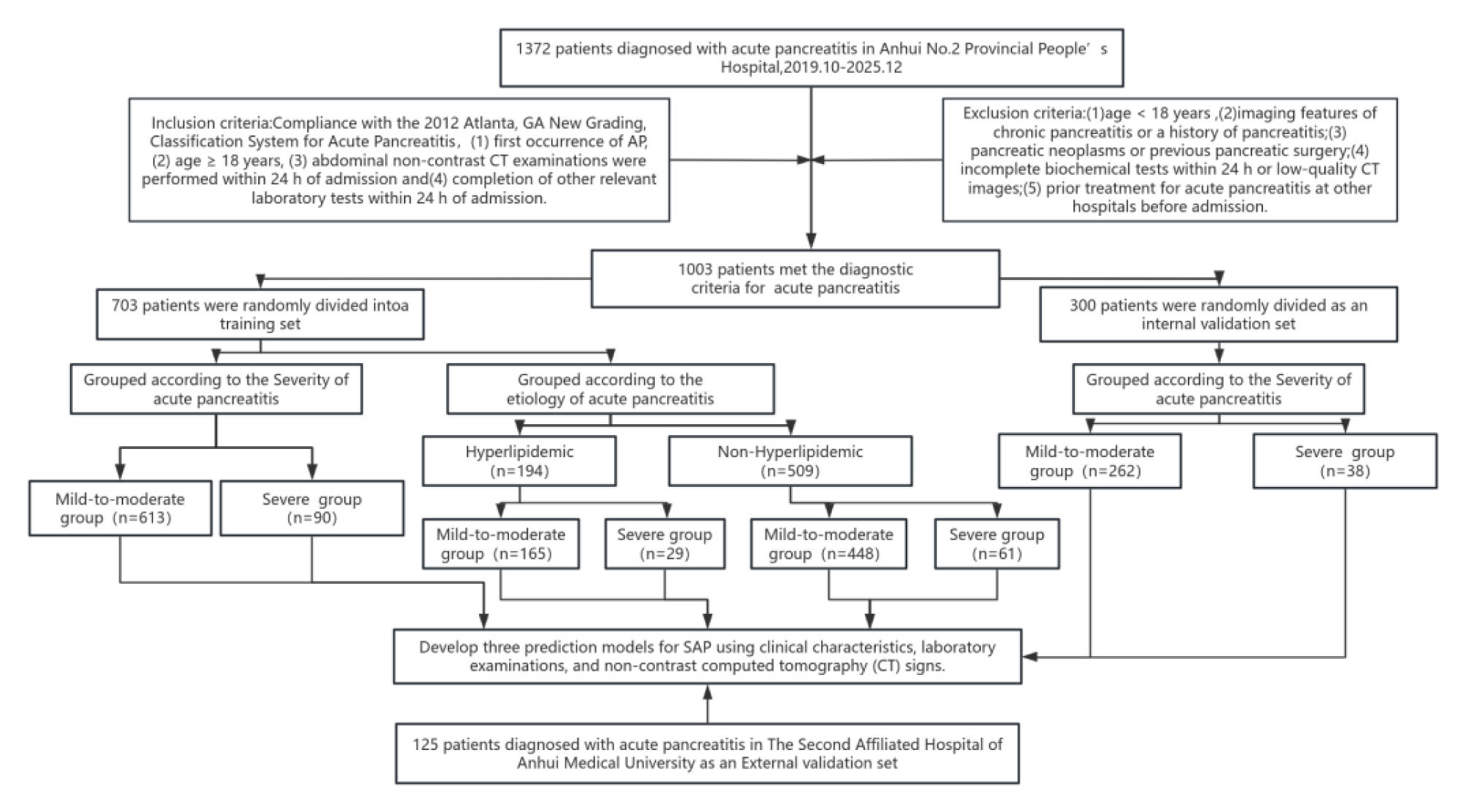

Supplement: Supplementary Figure S1 — Study inclusion flowchart. [file Image_1.tif]

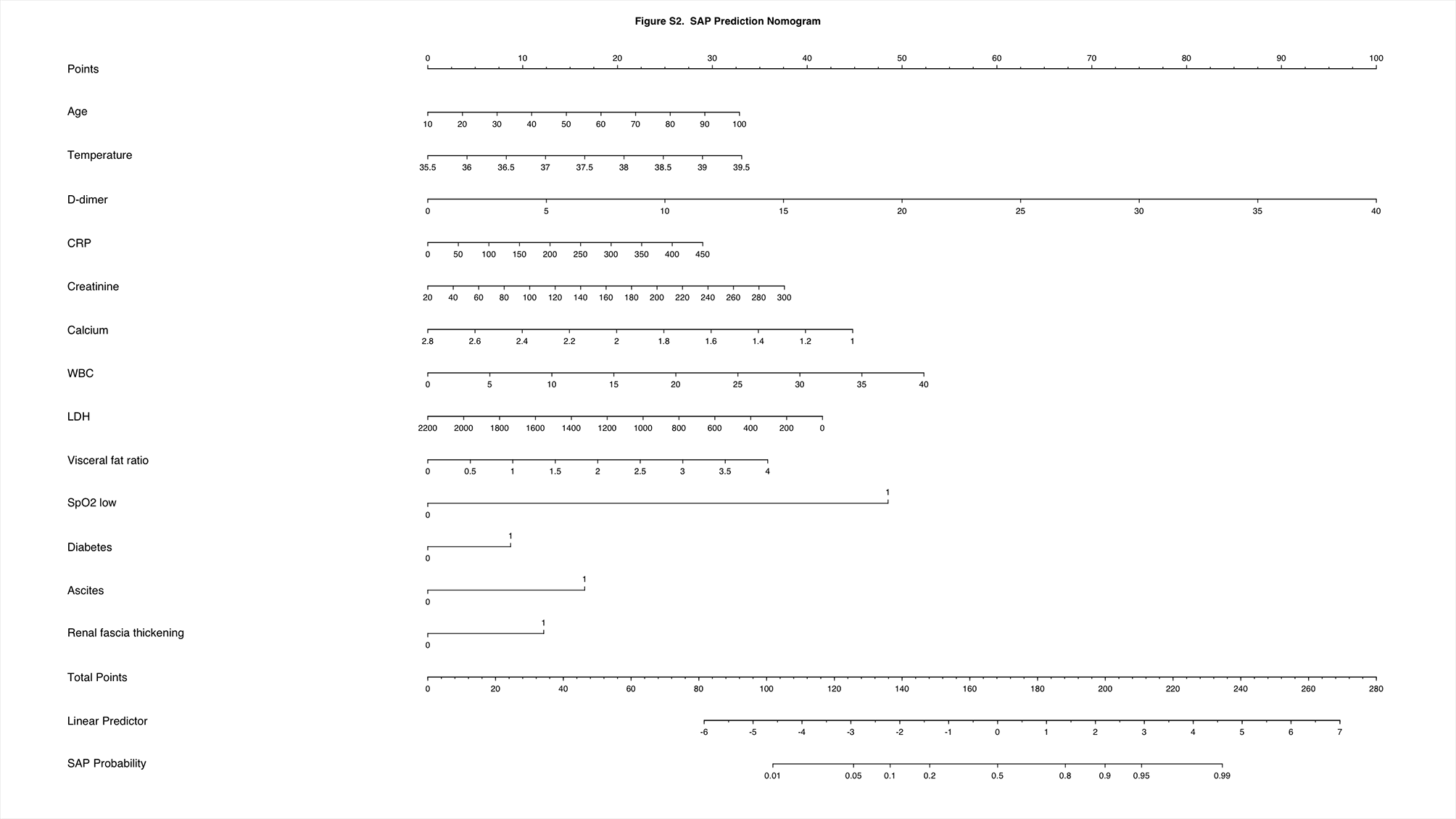

Supplement: Supplementary Figure S2 — Nomogram for severe pancreatitis among all pancreatitis patients. MAP, mean arterial pressure; CRP 24h, C-reactive protein within 24 hours after admission. [file Image_2.tif]

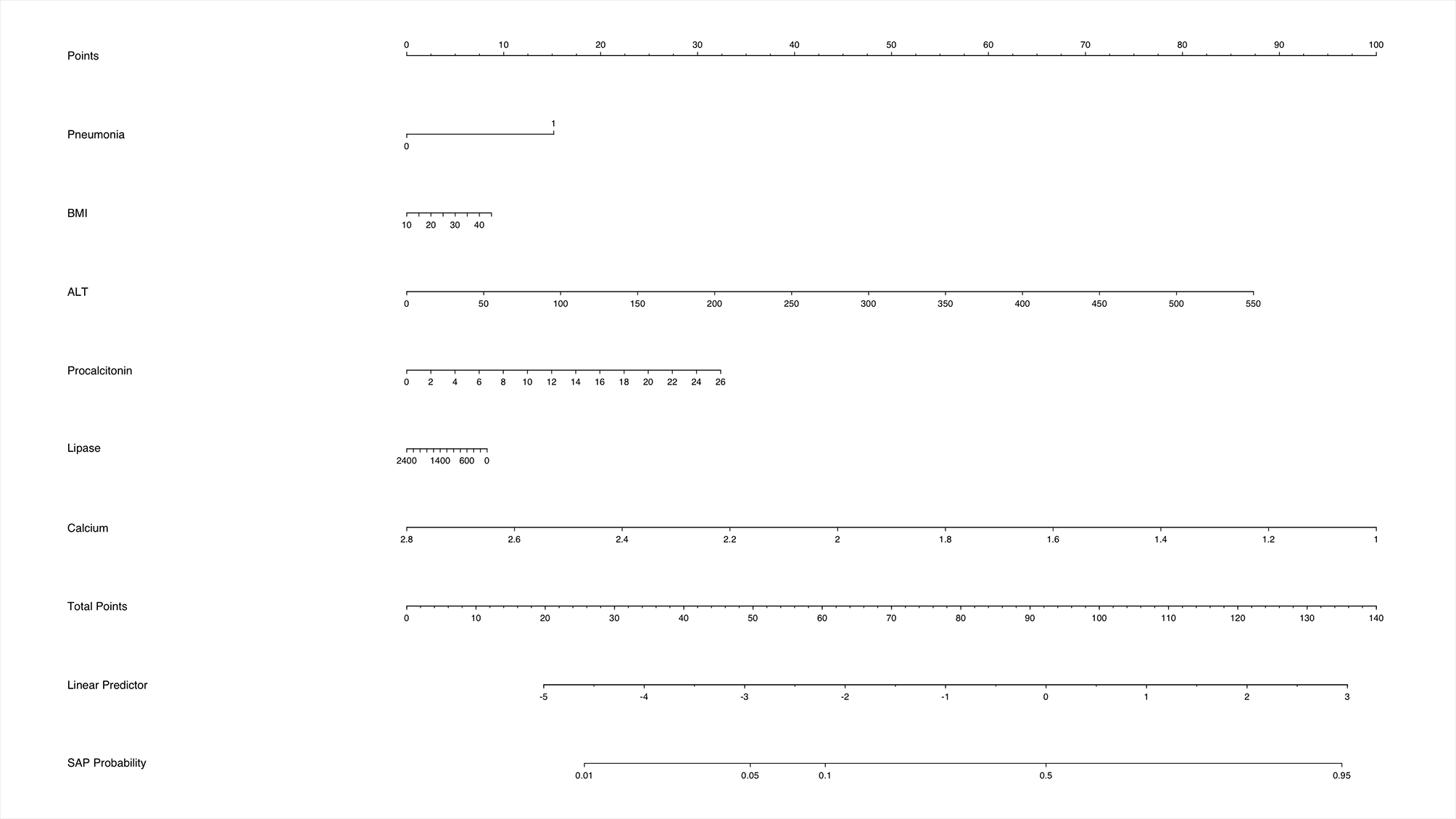

Supplement: Supplementary Figure S3 — Nomogram for severe pancreatitis among hyperlipidemic acute pancreatitis patients. MAP, mean arterial pressure; BMI, body mass index; ALT, alanine aminotransferase. [file Image_3.tif]

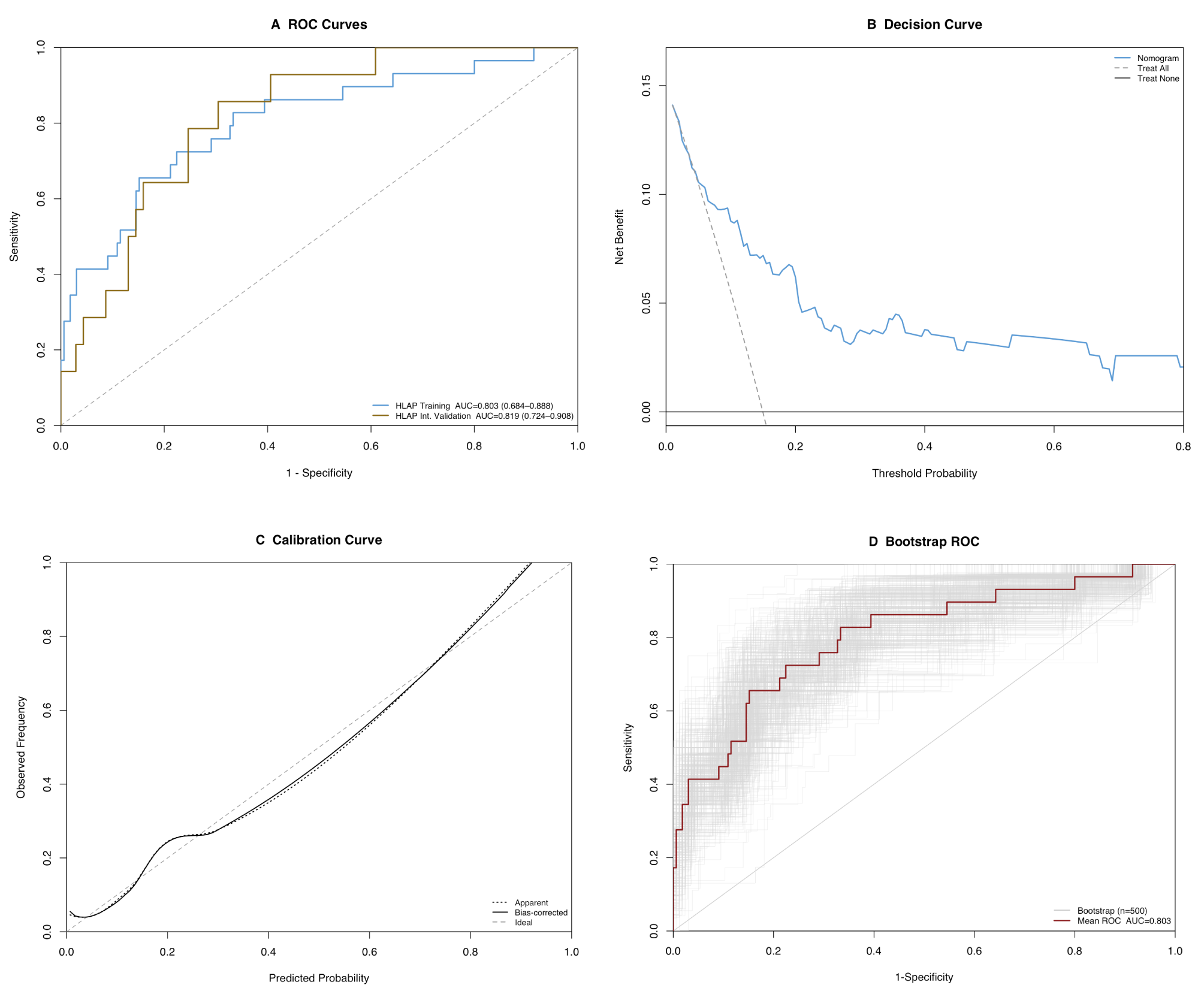

Supplement: Supplementary Figure S4 — Model performance among hyperlipidemic acute pancreatitis patients. (A) ROC curve; (B) DCA curve; (C) calibration curve; (D) ROC curve in bootstrap internal validation. [file Image_4.tif]

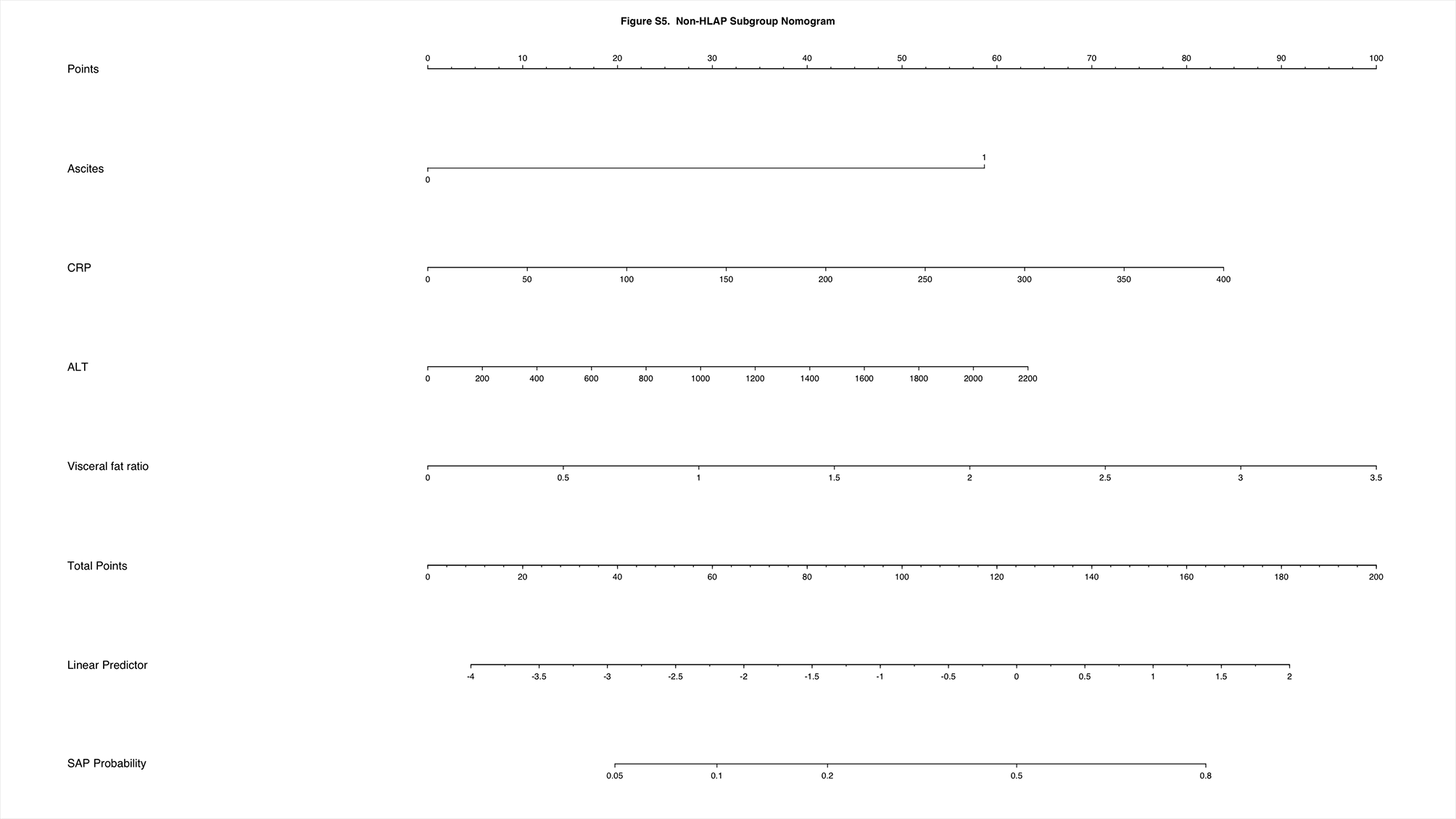

Supplement: Supplementary Figure S5 — Nomogram for severe pancreatitis among non-hyperlipidemic acute pancreatitis patients. BMI, body mass index; CRP 24h, C-reactive-protein within 24 h after admission; ALT, alanine aminotransferase. [file Image_5.tif]

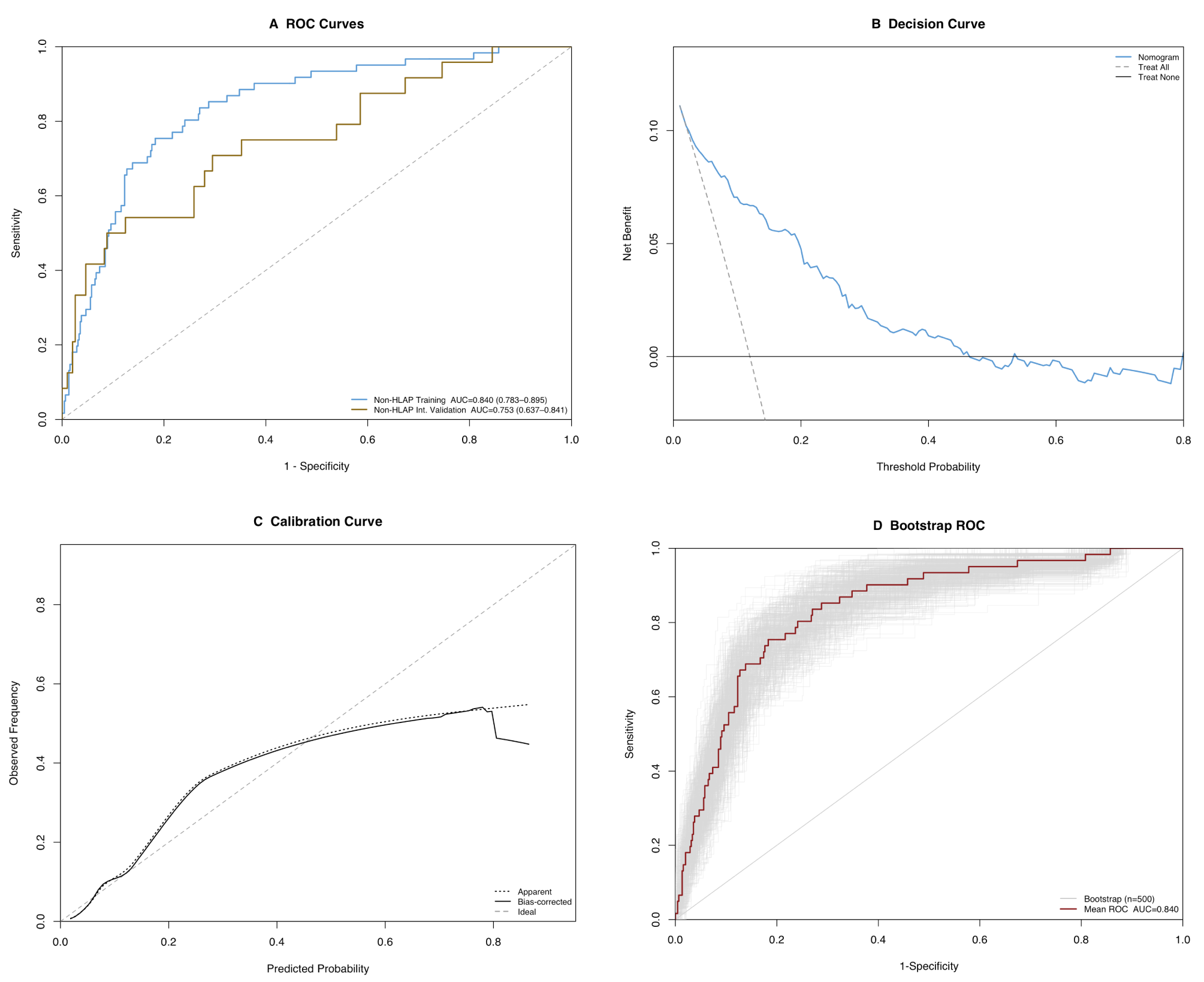

Supplement: Supplementary Figure S6 — Model performance and evaluation with non-hyperlipidemic acute pancreatitis patients. (A) ROC curve; (B) DCA curve; (C) calibration curve; (D) ROC curve in bootstrap internal validation. [file Image_6.tif]
