# Supplementary material for: Development of a prediction method for severe pancreatitis using a nomogram
Source: Front Med (Lausanne). 2026 May 7;13:1737122. doi: 10.3389/fmed.2026.1737122 (PMC13190413; doi:10.3389/fmed.2026.1737122)
Supplement: Supplementary Table S1 — Comparison of the training, internal validation, and external validation sets. [file Table_1.docx]

**Table S1.** Comparison of the training, internal validation, and external validation sets.

|  | External Validation | Internal Validation | Training | P |
| --- | --- | --- | --- | --- |
| n | 125 | 300 | 703 |  |
| Age (median [IQR]) | 42.00 [34.00, 57.00] | 43.00 [34.00, 57.00] | 46.00 [35.00, 59.50] | 0.066 |
| Sex (female) | 41 (32.8) | 124 (41.3) | 275 (39.1) | 0.258 |
| BMI (median [IQR]) | 22.70 [21.70, 23.60] | 24.97 [22.64, 27.28] | 24.65 [22.59, 27.04] | <0.001 |
| SBP (median [IQR]) | 108.00 [101.00, 114.00] | 132.00 [120.00, 145.00] | 130.00 [118.00, 144.00] | <0.001 |
| DBP (median [IQR]) | 71.00 [66.00, 74.00] | 82.00 [75.00, 91.00] | 82.00 [73.00, 91.00] | <0.001 |
| MAP (median [IQR]) | 83.00 [80.00, 85.67] | 99.67 [90.58, 107.67] | 98.00 [89.50, 107.67] | <0.001 |
| HR (median [IQR]) | 73.00 [68.00, 78.00] | 85.00 [75.00, 100.00] | 85.00 [75.00, 100.00] | <0.001 |
| temp (median [IQR]) | 36.50 [36.30, 36.80] | 36.50 [36.40, 36.60] | 36.50 [36.40, 36.60] | 0.668 |
| onset_h (median [IQR]) | 12.00 [6.00, 24.00] | 12.00 [7.00, 24.00] | 14.00 [7.00, 48.00] | 0.844 |
| PCT (median [IQR]) | 0.18 [0.11, 1.14] | 0.15 [0.07, 0.32] | 0.15 [0.07, 0.34] | <0.001 |
| lipase (median [IQR]) | 522.00 [223.00, 1808.00] | 223.00 [76.41, 596.30] | 223.00 [87.25, 575.60] | <0.001 |
| amylase (median [IQR]) | 212.00 [104.00, 650.00] | 150.00 [69.75, 523.25] | 144.00 [71.00, 577.50] | 0.061 |
| D_dimer (median [IQR]) | 1.09 [0.50, 2.56] | 1.14 [0.70, 2.16] | 1.14 [0.56, 1.99] | 0.272 |
| CRP (median [IQR]) | 17.79 [5.40, 57.79] | 41.29 [14.03, 115.11] | 41.29 [13.31, 102.91] | <0.001 |
| TG (median [IQR]) | 3.32 [1.12, 11.32] | 2.10 [1.08, 6.89] | 2.08 [1.02, 6.68] | 0.070 |
| creatinine (median [IQR]) | 65.00 [52.00, 80.00] | 60.00 [49.75, 73.00] | 61.00 [50.00, 75.00] | 0.023 |
| BUN (median [IQR]) | 4.74 [3.61, 5.73] | 4.00 [3.00, 5.60] | 4.10 [3.10, 5.45] | 0.011 |
| calcium (median [IQR]) | 2.17 [2.05, 2.30] | 2.17 [2.03, 2.27] | 2.16 [2.06, 2.26] | 0.499 |
| WBC (median [IQR]) | 10.90 [8.88, 13.57] | 9.51 [6.48, 12.91] | 9.63 [7.01, 12.94] | 0.002 |
| neutrophil (median [IQR]) | 65.80 [9.50, 77.60] | 7.64 [4.74, 10.63] | 7.80 [5.00, 10.95] | <0.001 |
| lymphocyte (median [IQR]) | 6.70 [1.56, 16.80] | 1.20 [0.84, 1.60] | 1.25 [0.85, 1.71] | <0.001 |
| NLR (median [IQR]) | 5.38 [3.90, 9.68] | 6.44 [3.27, 10.14] | 6.14 [3.38, 10.49] | 0.873 |
| neutrophil_ratio (median [IQR]) | 79.90 [71.20, 87.50] | 80.25 [69.38, 86.03] | 79.30 [69.00, 86.35] | 0.406 |
| LDH (median [IQR]) | 198.00 [156.00, 245.00] | 202.50 [151.00, 277.00] | 198.00 [160.07, 259.87] | 0.924 |
| ALT (median [IQR]) | 49.00 [31.00, 106.00] | 36.00 [21.00, 74.25] | 35.00 [20.00, 89.00] | 0.001 |
| glucose (median [IQR]) | 6.76 [5.73, 9.36] | 7.29 [5.73, 10.53] | 7.36 [5.82, 10.57] | 0.282 |
| RPIS (median [IQR]) | 1.00 [1.00, 1.00] | 1.00 [1.00, 2.00] | 1.00 [1.00, 1.00] | 0.001 |
| VFR (median [IQR]) | 0.84 [0.59, 1.08] | 0.84 [0.64, 1.10] | 0.87 [0.67, 1.20] | 0.121 |
| SpO2_low (mean (SD)) | 0.02 (0.13) | 0.01 (0.11) | 0.02 (0.13) | 0.911 |
| smoking = 1 (%) | 10 (8.0) | 113 (37.7) | 258 (36.7) | <0.001 |
| drinking = 1 (%) | 5 (4.0) | 129 (43.0) | 264 (37.6) | <0.001 |
| diabetes = 1 (%) | 23 (18.4) | 121 (40.3) | 278 (39.5) | <0.001 |
| hypertension = 1 (%) | 4 (3.2) | 127 (42.3) | 295 (42.0) | <0.001 |
| HTG_med_hx (mean (SD)) | 0.00 (0.00) | 0.33 (0.47) | 0.33 (0.47) | <0.001 |
| abd_distension (mean (SD)) | 0.84 (0.37) | 0.40 (0.49) | 0.43 (0.50) | <0.001 |
| abd_tenderness (mean (SD)) | 0.89 (0.32) | 0.74 (0.44) | 0.77 (0.42) | 0.003 |
| ileus (mean (SD)) | 0.01 (0.09) | 0.00 (0.06) | 0.01 (0.08) | 0.814 |
| pneumonia = 1 (%) | 12 (9.6) | 147 (49.0) | 331 (47.1) | <0.001 |
| pleural_effusion = 1 (%) | 10 (8.0) | 49 (16.3) | 124 (17.6) | 0.027 |
| ascites = 1 (%) | 14 (11.2) | 50 (16.7) | 110 (15.6) | 0.351 |
| fatty_liver = 1 (%) | 66 (52.8) | 177 (59.0) | 409 (58.2) | 0.472 |
| cholecystitis = 1 (%) | 34 (27.2) | 91 (30.3) | 213 (30.3) | 0.774 |
| gallstones = 1 (%) | 33 (26.4) | 56 (18.7) | 140 (19.9) | 0.180 |
| renal_fascia = 1 (%) | 79 (63.2) | 207 (69.0) | 480 (68.3) | 0.477 |
| cholecystectomy (mean (SD)) | 0.09 (0.28) | 0.08 (0.27) | 0.08 (0.26) | 0.880 |

There were no significant difference between the internal validation and training sets.

**Table S2. Multicollinearity assessmen**t

| **Model** | **Variable** | **VIF** | **Pass** |
| --- | --- | --- | --- |
| Main Model | age | 1.072 | TRUE |
| Main Model | temp | 1.018 | TRUE |
| Main Model | D_dimer | 1.202 | TRUE |
| Main Model | CRP | 1.25 | TRUE |
| Main Model | creatinine | 1.066 | TRUE |
| Main Model | calcium | 1.131 | TRUE |
| Main Model | WBC | 1.089 | TRUE |
| Main Model | LDH | 1.091 | TRUE |
| Main Model | VFR | 1.075 | TRUE |
| Main Model | SpO2_low | 1.039 | TRUE |
| Main Model | diabetes | 1.027 | TRUE |
| Main Model | ascites | 1.174 | TRUE |
| Main Model | renal_fascia | 1.088 | TRUE |
| Non-HLAP | ascites | 1.051 | TRUE |
| Non-HLAP | CRP | 1.101 | TRUE |
| Non-HLAP | ALT | 1.027 | TRUE |
| Non-HLAP | VFR | 1.044 | TRUE |

VIF < 5 indicates acceptable multicollinearity. All values < 2. CRP, C-reactive protein; LDH, lactate dehydrogenase; VFR, visceral fat ratio.

**Table S3. Univariable and multivariable analysis for the development of severe pancreatitis in hyperlipidemic acute pancreatitis patients.**

| **Variables** | **Univariable** | | | | **Multivariable** | | | |
| --- | --- | --- | --- | --- | --- | --- | --- | --- |
|  | **β** | **P** | **OR** | **OR (95%CI)** | **β** | **P** | **OR** | **OR (95%CI)** |
| ALT | 0.006 | 0.0542 | 1.006 | 1.006 (1–1.018) | 0.009 | 0.0096 | 1.009 | 1.009 (1.002–1.026) |
| BMI | 0.0176 | 0.7275 | 1.018 | 1.018 (0.919–1.119) | 0.0171 | 0.7715 | 1.017 | 1.017 (0.904–1.14) |
| Serum calcium | -4.3593 | 0 | 0.013 | 0.013 (0.002–0.077) | -3.8824 | 1e-04 | 0.021 | 0.021 (0.002–0.151) |
| Lipase | 7e-04 | 0.1732 | 1.001 | 1.001 (1–1.002) | -3e-04 | 0.6724 | 1 | 1 (0.998–1.001) |
| Procalcitonin | 0.1339 | 0.0329 | 1.143 | 1.143 (1.011–1.41) | 0.0317 | 0.6893 | 1.032 | 1.032 (0.899–1.408) |
| Pneumonia | 1.4041 | 6e-04 | 4.072 | 4.072 (1.813–9.753) | 1.0975 | 0.0173 | 2.997 | 2.997 (1.212–7.877) |

ALT, alanine aminotransferase; BMI, body mass index; BUN, blood urea nitrogen; CI: Confidence Interval; CRP, C-reactive protein; LDH, lactate dehydrogenase; MAP, mean arterial pressure; NLR, Neutrophil-to-Lymphocyte Ratio; OR: Odds Ratio; WBC, white blood cell.

**Table S4. Univariable and multivariable analysis for the development of severe pancreatitis in non-hyperlipidemic acute pancreatitis patients.**

| **Variables** | **Univariable** | | | | **Multivariable** | | | |
| --- | --- | --- | --- | --- | --- | --- | --- | --- |
|  | **β** | **P** | **OR** | **OR (95%CI)** | **β** | **P** | **OR** | **OR (95%CI)** |
| ALT | -4e-04 | 0.6871 | 1 | 1 (0.998–1.001) | 0.001 | 0.2413 | 1.001 | 1.001 (0.999–1.003) |
| Ascites | 2.1517 | 0 | 8.599 | 8.599 (4.823–15.333) | 2.0394 | 0 | 7.686 | 7.686 (4.106–14.387) |
| CRP | 0.0095 | 0 | 1.01 | 1.01 (1.006–1.013) | 0.0073 | 1e-04 | 1.007 | 1.007 (1.004–1.011) |
| VFR | 0.9818 | 1e-04 | 2.669 | 2.669 (1.63–4.372) | 0.993 | 7e-04 | 2.699 | 2.699 (1.525–4.778) |

ALT, alanine aminotransferase; BMI, body mass index; BUN, blood urea nitrogen; CI: Confidence Interval; CRP, C-reactive protein; LDH, lactate dehydrogenase; MAP, mean arterial pressure; NLR, Neutrophil-to-Lymphocyte Ratio; OR: Odds Ratio; WBC, white blood cell.
